# Defining the Tumor Microenvironment by Integration of Immunohistochemistry and Extracellular Matrix Targeted Imaging Mass Spectrometry

**DOI:** 10.3390/cancers13174419

**Published:** 2021-09-01

**Authors:** Denys Rujchanarong, Julia Lefler, Janet E. Saunders, Sarah Pippin, Laura Spruill, Jennifer R. Bethard, Lauren E. Ball, Anand S. Mehta, Richard R. Drake, Michael C. Ostrowski, Peggi M. Angel

**Affiliations:** 1 Department of Cell and Molecular Pharmacology & Experimental Therapeutics, Bruker-MUSC Center of Excellence, Clinical Glycomics, Medical University of South Carolina, Charleston, SC 29425, USA; rujchana@musc.edu (D.R.); saundej@musc.edu (J.E.S.); pippins@glycopath.com (S.P.); bethard@musc.edu (J.R.B.); ballle@musc.edu (L.E.B.); mehtaa@musc.edu (A.S.M.); draker@musc.edu (R.R.D.); 2 Department of Biochemistry and Molecular Biology, Medical University of South Carolina, Charleston, SC 29425, USA; lefler@musc.edu (J.L.); ostrowsk@musc.edu (M.C.O.); 3 Department of Pathology and Laboratory Medicine, Medical University of South Carolina, Charleston, SC 29425, USA; spruill@musc.edu

**Keywords:** imaging mass spectrometry (IMS), immunohistochemistry (IHC), PTEN, stroma, ECM, tumor microenvironment, breast cancer

## Abstract

**Simple Summary:**

Extracellular matrix within the tumor microenvironment influences signaling, controls molecular diffusion of nutrients and growth factors, alters immunogenicity, and contributes to disease progression and therapeutic response. ECM is secreted by multiple cell types, including tumor cells, fibroblasts, and immune cells, yet there are limited approaches that link the cell type to the ECM proteins within the heterogeneous tumor microenvironment. Here, we show that integrating immunohistochemistry (IHC) with extracellular matrix (ECM) imaging mass spectrometry allows ECM proteomic profiling based on patterns of diverse cell types and proteins in tissue. The developed approach is demonstrated using phosphatase and tensin homolog (PTEN) staining and ECM imaging/proteomics on the same tissue sections in normal breast and in a tissue microarray of breast tumor and normal adjacent tissue. The data suggests that PTEN expression in tumor and in normal adjacent tissue may be associated with different collagen types and regulation by post-translational sites of modification.

**Abstract:**

Breast stroma plays a significant role in breast cancer risk and progression yet remains poorly understood. In breast stroma, collagen is the most abundantly expressed protein and its increased deposition and alignment contributes to progression and poor prognosis. Collagen post-translation modifications such as hydroxylated-proline (HYP) control deposition and stromal organization. The clinical relevance of collagen HYP site modifications in cancer processes remains undefined due to technical issues accessing collagen from formalin-fixed, paraffin-embedded (FFPE) tissues. We previously developed a targeted approach for investigating collagen and other extracellular matrix proteins from FFPE tissue. Here, we hypothesized that immunohistochemistry staining for fibroblastic markers would not interfere with targeted detection of collagen stroma peptides and could reveal peptide regulation influenced by specific cell types. Our initial work demonstrated that stromal peptide peak intensities when using MALD-IMS following IHC staining (αSMA, FAP, P4HA3 and PTEN) were comparable to serial sections of nonstained tissue. Analysis of histology-directed IMS using PTEN on breast tissues and TMAs revealed heterogeneous PTEN staining patterns and suggestive roles in stromal protein regulation. This study sets the foundation for investigations of target cell types and their unique contribution to collagen regulation within extracellular matrix niches.

## 1. Introduction

Breast cancer affects one in eight women in the United States; in 2016, for every 100,000 women, 124 females reported to have breast cancer and of those, 20 women died [1,2]. Among women in the US, breast cancer is the second leading cause of cancer death, following lung cancer [2]. Progress has been made in early detection of breast cancer leading to optimistic outcomes with a five-year survival rate of 99% for localized breast cancers [3]. However, once the tumor has metastasized, treatments become limited and the five-year survival rate significantly drops to about 27% [3]. Emerging research have found that breast stroma plays a crucial role in breast cancer development, progression and outcome [3], yet the breast stroma remains poorly understood.

The crosstalk between stromal cells and the tumor stroma involved in cancer progression has been widely recognized as related to outcome and therapeutic resistance [4,5,6,7]. Tissue stroma includes extracellular matrix connective tissue, blood vessels, and fibroblasts, somatic, and immune cells that maintain tissue homeostasis [8]. The interaction between tumors and their stroma determines the tumor phenotype and is critical for tumor survival and growth [8,9]. Tumors have been described as “a wound that never heals” as cancer stroma formation and maintenance mimics normal tissue undergoing wound healing eventually creating densely collagenous scar-like connective tissue [7,8,9,10]. Breast density, defined by higher proportion of stromal and epithelial cells, is significantly correlated with increased collagen deposition [5,7,11,12,13] and contributes to breast cancer risk and progression [3,5,7,14,15]. Once the tumor has developed, collagen realignment at the leading edge of the tumor border aids tumor growth and is predictive of patient survival [13,14].

Previous studies have associated the stromal reorganization involving stiffened collagen with higher probability of metastasis [3,15]. Collagens are a large family of fibrous proteins with triple helical structures that play important roles in scaffolding, cell adhesion and migration, tissue morphogenesis, and cancer [16]. Changes in collagen within the tumor and stromal boundary have been characterized as mammary carcinoma progression markers known as tumor associated collagen signatures (TACS) [3,13]. There are three known types of TACS: TACS-1, TACS-2, and TACS-3, which consist of increased collagen deposition localized near the tumor lesion, straighter collagen fibers that are aligned parallel to the tumor boundary, and collagen reorientation such that the collagen fibers are bundled and aligned perpendicularly to the tumor boundary, respectively [13]. Cancer-activated fibroblasts have been earmarked as generating the invasive collagen “highways” that aid in metastasis [15,17].

Cancer stroma plays a critical role in influencing tumors and is affected by tumor suppressors such as phosphatase and tensin homolog deleted on chromosome 10 (PTEN); stromal depletion of PTEN has been shown to increase tumorigenesis [15,18]. Tumor suppressor PTEN is located in different subcellular locations of cells and may be secreted into stroma [19]. PTEN can antagonize pathways, particularly the phosphoinositide 3-kinase (PI3K) pathway that is involved in cell proliferation, differentiation, growth, and survival [20,21,22], making PTEN a critical component in cancer signaling [23]. Mechanistically, PTEN works to dephosphorylate phosphatidylinositol-3,4,5-trisphosphate (PIP3) to phosphatidylinositol-4,5-bisphosphate (PIP2), negatively regulating the PI3K signaling pathway. Decreases in PTEN result in RAS activation that enhance migration, invasion and increase AKT2 phosphorylation [24,25,26,27,28]. PTEN is commonly lost in sporadic cancers including prostate, lung, endometrial, and breast [28,29,30,31].Loss of PTEN activity occurs through mutations, deletion, or silencing and is found in germlines with a predisposition to cancer. Interestingly, PTEN decreases have been associated with regulating matrix remodeling leading to stromal expansion and tumor progression [15]. In breast cancer, PTEN depletion has been shown to result in increased collagen alignment parallel to the mammary ducts compared to normal tissue [15]. PTEN is expressed in normal breast tissue and decreases result in oncogenic realignment of collagen in the stroma [15,32]. Its loss in tissues has been proposed as a biomarker in diseases such as triple-negative breast cancer [20,23,33].

PTEN localizes to the cell nucleus and is heterogeneously expressed by the stromal cells that may have different roles in regulating collagen that facilitates oncogenic signaling. In this study, we hypothesized that immunohistochemistry could be combined with extracellular matrix targeted imaging proteomics [34,35,36,37] to understand collagen regulation related to PTEN positive or negative cells from normal breast tissue or the breast tumor microenvironment. ECM imaging was completed after staining and documenting tissue distribution of breast cancer driven cell markers, fibroblast activated protein (FAP), alpha smooth muscle actin (αSMA), and prolyl-4-hydroxylase subunit alpha 3 (P4HA3). Detection of collagen peptides on IHC-stained tissue was found to fall within the 10% variation detected in serial sections of breast tumor. Evaluation of PTEN-scored normal breast tissue from reductive mammoplasty by both ECM imaging proteomics and chromatography coupled to tandem mass spectrometry suggested that very specific sites of collagen hydroxylated prolines may be influenced by PTEN expression levels [38]. Further evaluation of a PTEN-stained tissue microarray of invasive ductal carcinoma, adjacent to tumor, and normal adjacent further demonstrated site-specific proline hydroxylation may differ within the tumor microenvironment compared to normal adjacent tissue. This method increases our capacity to understand extracellular matrix niches produced by specific cell types from both the normal tissue microenvironment to tumor microenvironment.

## 2. Materials and Methods

### 2.1. Overview

Collagenase type III (COLase3) (*Clostridium histolyticum*) was obtained from Worthington (Lakewood, NJ, USA). PNGaseF PRIME™ was obtained from N-Zyme Scientific (Doylestown, PA, USA). Sigma Aldrich (St. Louis, MO, USA) was the source used for acetonitrile, α-cyano-4-hydroxycinnamic acid (CHCA), trifluoroacetic acid (TFA), ammonium bicarbonate, ammonium phosphate monobasic, and calcium chloride. Dewaxing (xylenes, 200-proof ethanol, methanol) solvents and high-performance liquid chromatography (HPLC)-grade water were purchased from Fisher Scientific (Pittsburgh, PA, USA).

### 2.2. Tissues

Liver and breast tissue use was approved by the Medical University of South Carolina Institutional Review Board. Liver tissue and breast tissue sections were obtained from MUSC Hollings Cancer Center (HCC) Biorepository. Technical replicates of breast cancer tissues (*n* = 5) were used to investigate breast tissue heterogeneity among serial sections. Groups of technical replicates of liver (*n* = 3) and breast tissue slides (*n* = 3) were used for different stains that were then compared to their nonstained counterparts (*n* = 3). Certain PTEN-stained normal breast tissues categorized as low PTEN (*n* = 3; biological replicates) and high PTEN (*n* = 4; biological replicates) were archival tissues from Michael Ostrowski [38]. Two tissue microarrays (US Biomax, Rockville, MD, USA) created in 2014 and graded by the WHO 2012 guidelines were composed of 134 cases and 144 cores with invasive ductal carcinoma as the malignant tumor, adjacent to tumor (AT) and normal adjacent to tumor.

### 2.3. Immunohistochemistry (IHC) Tissue Preparation

Formalin-fixed paraffin embedded (FFPE) tissues were heated, dewaxed, and antigen-retrieved at pH 6 using sodium citrate buffer following validated protocols (proteinatlas.org accessed on August 2020) [39]. Breast tissues were stained using nuclear Mayer’s hematoxylin (Electron Microscopy Sciences, Hatfield, PA, USA), hematoxylin and eosin (H&E, Cancer Diagnostics, Inc. and FisherBrand, Pittsburgh, PA, USA), prolyl 4-hydroxylase subunit-alpha 3 (P4HA3, Novus Biologicals, LLC, Centennial, CO, USA), fibroblast activation protein (FAP, Novus Biologicals, LLC, Centennial, CO, USA), alpha smooth muscle actin (α-SMA, Novus Biologicals, LLC, Centennial, CO, USA) and phosphatase and tensin homolog (PTEN, Cell Signaling Technology, Inc., Boston, MA, USA). For different tissue staining, Mayer’s hematoxylin was incubated for 1 min, hematoxylin was incubated for 2 min and eosin for 1 min. P4HA3 (1:100), FAP (1:100) and α-SMA (1:1000) were incubated overnight and PTEN (1:200) was autostained by the Cancer Tissue and Pathology Shared Resource at Emory Winship Cancer Institute. Stains were digitally scanned at 40× (Nanozoomer, Hamamatsu; Shizuoka, Japan). H-scores for PTEN in reductive mammoplasty were calculated using the relative intensity of staining and number of stained cells as previously described [38].

PTEN staining (1:200) of human breast TMAs was quantified using Fiji ImageJ (Java 8, Bethesda, MD, USA) [40]. The files of the TMAs scanned were exported from Nanozoom software as a JPEG file. The JPEG was opened in Fiji ImageJ and each punch biopsy was saved as an individual tiff file, by selecting the area of the biopsy and then “Duplicate” to make a single file. The file was color-deconvoluted (Image > Color > Color Deconvolution) and the vector DAB, Color 2 (brown color) file was used to report the mean area of stain per core after color inversion [41,42].

### 2.4. Imaging Mass Spectrometry (IMS) Preparation

Tissue was prepared as previously with minor modifications [34,35,43,44]. Previously stained tissues were incubated in xylene for 24–72 h to remove coverslips. Tissues were then destained with xylene and ethanol washes, heat-induced epitope retrieved with 10 mM citraconic buffer, pH 3 for optimal deglycosylation. An automated sprayer was used to apply PNGaseF PRIME enzyme using an automated sprayer (M3 TM-Sprayer, HTXImaging, Chapel Hill, NC, USA) to remove N-glycans [45]. Tissues were washed to remove N-glycans [46], then heat-induced epitope retrieved using 10 mM Tris buffer, 1 mM CaCl_2_, pH 9 for optimal collagenase access. Tissues were sprayed with COLase3 enzyme using an automated sprayer with parameters of 45 °C, 10 psi, 25 µL/min, 1200 velocity, and 15 passes. Tissues were digested in ≥80% relative humidity at 37.5 °C for 5 h followed by spraying CHCA matrix (7 mg/mL CHCA in 50% acetonitrile, 1% trifluoracetic acid). For matrix spraying, parameters were 79 °C, 10 psi, 100 µL/min, 1300 velocity and 10 passes. To limit matrix clusters, slides were rapidly dipped in cold 5 mM ammonium phosphate monobasic and dried in desiccator prior to IMS data acquisition. Control tissues were processed through the same methods minus the staining components following previous protocols.

### 2.5. IMS Data Acquisition

Serial breast cancer tissue sections were analyzed by a Fourier Transform Ion Cyclotron Resonance mass spectrometer (FT-ICR) (solariX Legacy 7.0 Tesla, Bruker, Bremen, Germany) equipped with a matrix-assisted laser desorption/ionization (MALDI) source using positive ion mode over *m*/*z* range of 700–2500. Laser settings used were 200 laser shots per pixel with a step size of 125 to 200-µm between pixels. Time of flight was set to 1.050 ms and transient length was 0.8389 s. The [Glu1]-Fibrinopeptide B human (*m*/*z* 1570.6768; Sigma) was used as an internal standard and lockmass for peptide IMS studies.

All images were visualized in FlexImaging v5.0 and analyzed by SCiLS Lab software 2021b Premium 3D Build 9.01.12514 (Bruker Scientific, LLC, Bremen, Germany) with linear interpolation without denoising normalized to total ion count. SCiLS Lab settings for visualizing the peak intensities were set to a 99% quantile. Exported peak intensities were transformed using natural log. Statistical significance of all extracted peak intensities compared between regions were determined using exact *p*-values calculated by student *t*-test using GraphPad version 9.0.2 (San Diego, CA, USA).

### 2.6. Proteomics

After imaging, matrix was removed from the breast tissue sections using ethanol washes as previously described [46]. Each tissue section was scraped into individual centrifuge tubes and digested with COLase3 overnight [35]. A C18 StageTip (Thermo Fisher Scientific, San Jose, CA, USA) was used to desalt peptides prior to loading onto a trap column for separation. Peptides were analyzed by an Orbitrap Fusion Lumos Tribrid mass spectrometer (Thermo Fisher Scientific) with instrumental control software v. 4.2.28.14. A UHPLC (EASY nLC 1200 System, Thermo Fisher Scientific) was used to separate peptides on a C18 reverse phase column (Acclaim PepMap RSLC, 75 µm × 50 cm (C18, 2 µm, 100 Å), Thermo Fisher Scientific). A solvent gradient of 2–35% in 180 min at a flow rate of 300 nL/min was used for separation with Solvent A (0.1% FA); Solvent B (80% ACN/ 0.1% FA). A high-resolution (60,000) FTMS survey scan using a mass range of *m*/*z* 375–1575 was followed by tandem mass spectra (MS/MS) of the most intense precursors (charge states +1 through +5) with a cycle time of 3 s. An automatic gain control target value of 4.0 × 10^5^ was used for the survey MS scan. A precursor isolation window of 1.6 *m*/*z* was used with max injection time of 22 ms, and HCD collision energy of 35% with maximum; fragments were detected in the Orbitrap at a 15,000 resolution. Monoisotopic-precursor selection was set to “peptide” and Apex detection was not enabled. Precursors were dynamically excluded for 15 s and a mass tolerance of 10 ppm. Advanced peak determination was not enabled.

MS/MS data was searched using Mascot (Matrix Science, London, UK; version Mascot in Proteome Discoverer 1.4.0.288) and Sequest (Thermo Fisher Scientific, San Jose, CA, USA; version IseNode in Proteome Discoverer 1.4.0.288). A database was extracted from Uniprot for homo sapiens using keywords and gene ontology for extracellular matrix and collagen, dated 05052017 with 945 entries. This was appended with a contaminants database and concatenated with the reverse database of both the ECM proteome and the contaminants. Mascot and Sequest were set up to search assuming nonspecific enzymatic digestion. Deamidation of asparagine and glutamine and oxidation of lysine, methionine and proline were specified in Mascot and Sequest as variable modifications. Scaffold (version Scaffold_4.11.1, Proteome Software Inc., Portland, OR, USA) was used to report MS/MS-based peptide and protein identifications. Peptide identifications were accepted if they could be established at greater than 97.5% probability by the Peptide Prophet algorithm [47] Scaffold delta-mass correction. Protein identifications were accepted if they could be established at greater than 99.0% probability and contained at least two identified peptides. Protein probabilities were assigned by the Protein Prophet algorithm [48]. Proteins that contained similar peptides and could not be differentiated based on MS/MS analysis alone were grouped to satisfy the principles of parsimony. The false discovery rate for protein identification was 0.47%.

### 2.7. Statistical Analysis

GraphPad version 9.0.2 was used for the Anderson–Darling normal distribution test, the Spearman’s rank correlation coefficient (ρ) and receiver operating characteristic (ROC) curve analysis. The *p*-values < 0.05 were used to evaluate correlation between data. A ROUT analysis, a method based on the false discovery rate (FDR) with a maximum desired FDR of 1%, was used to identify outliers among peak intensities from stained data. Two peaks maximum were identified as outliers among 167 peaks from stained tissue data. Relative percent differences between stained and nonstained tissue were calculated by stained tissue peak intensity divided by nonstained tissue peak intensity. The Mann–Whitney U test was used to calculate *p*-values using GraphPad with *p*-values of <0.05 reported as significant results.

## 3. Results

### 3.1. Overview

**Figure 1:**
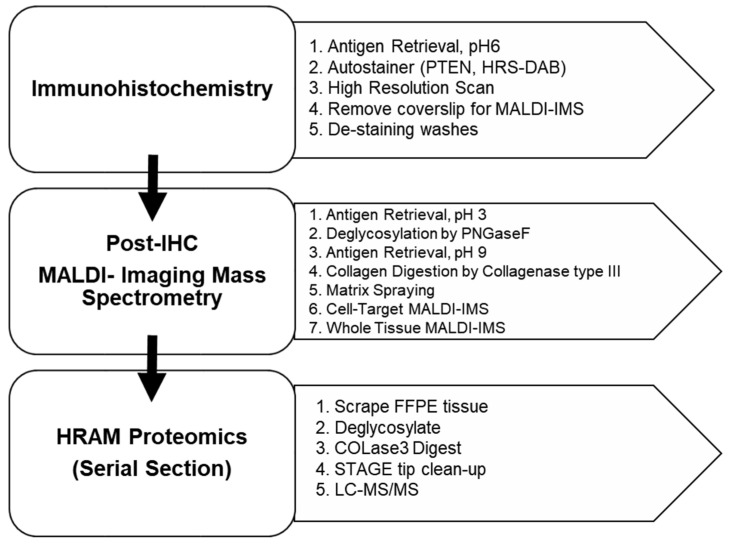
Workflow of integrated immunohistochemistry with MALDI-imaging mass spectrometry and high-resolution accurate mass (HRAM) proteomics.

To test that immunohistochemistry staining for cell markers would not interfere with detection of stromal peptides by IMS, a workflow was developed towards investigating imaging mass spectral data of previously immunohistochemistry-stained breast tissue (Figure 1). This approach allows an exact comparison of cell marker distribution compared to extracellular matrix patterns of the same tissue section. Relative percent change of H&E, nuclear stain, and antibody stains were compared to nonstained tissue to assess the method. Initial tests were done using hepatocellular carcinoma stained for trichrome, SOD2, and glypican. The approach was further developed for breast cancer studies comparing imaging mass spectra of peptide peaks between fibroblastic IHC stains (αSMA, FAP, P4HA3) and a control nonstained tissue of breast tumor. Imaging was followed by chromatography coupled to tandem mass spectrometry on the same tissue section to analyze peptides from normal breast tissue based on PTEN staining histology score (H-score) [37]. The study is concluded by evaluating a PTEN-stained tissue microarray of normal adjacent to tumor, adjacent (adenosis) and malignant breast tumor (invasive ductal carcinoma).

### 3.2. MALDI-IMS Following IHC Staining Allows Comparable Detection of ECM Peptides

To understand cell and extracellular matrix heterogeneity within the complex tissue microenvironment, we integrated the IHC workflow with tissue proteomic approaches (Figure 1). Previously, we demonstrated that targeting the stromal proteome on hematoxylin and eosin (H&E)-stained tissue by IMS produces nearly identical results when compared to nonstained tissue (Figure S1a). For IHC staining of cell markers, there are two main dyes used for detection: the chromophore label of the primary antibody and the contrasting nuclear dye. Preliminary testing done on hepatocellular carcinoma showed that only nuclear staining (no chromophore) compared to the nonstained tissue produced similar results with peak intensities varying by <6% (Figure S1b). Combined nuclear and chromophoric stains were tested on hepatocellular carcinoma (HCC) tissues using relevant antibodies. Staining done in triplicate on HCC tissue using stains trichrome, SOD2, and glypican independently demonstrated a <10% relative change when compared to a nonstained control tissue (Figure S1c–e). These data suggested that targeted ECM IMS after IHC was a promising approach towards understanding cell-specific ECM proteomes from the same tissue.

### 3.3. Nonstained Breast Tissues Reveal Minimal Intrinsic Peak Intensity Variation between Serial and Distant Sections

**Figure 2:**
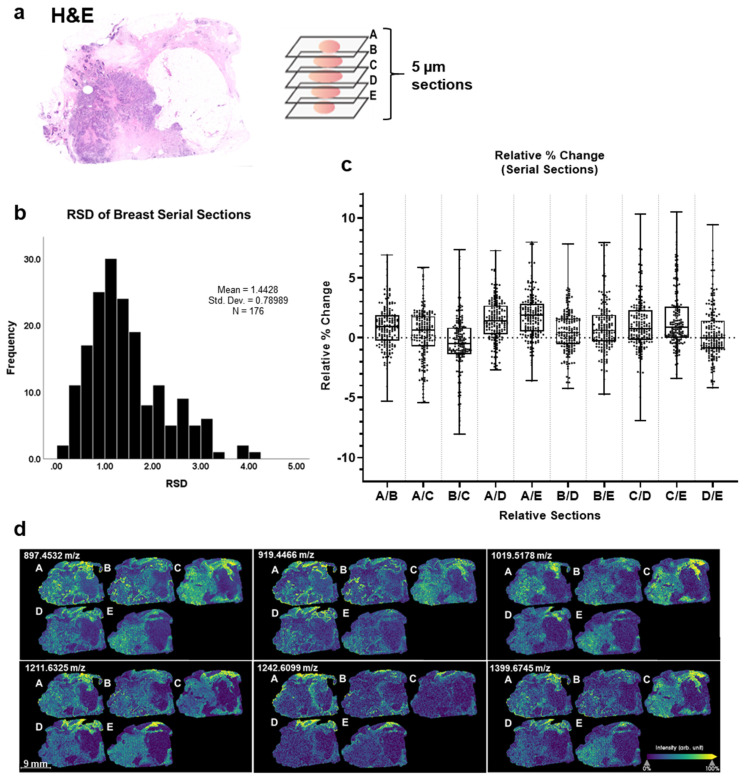
Breast tissues reveal minimal peak intensity variability between serial sections. (**a**) Breast cancer tissue stained with H&E with tumor region annotated. (**b**) Distribution of relative standard deviation of nonstained serial sections. (**c**) Percent change between 5 µm nonstained serial sections (A, B, C, D, and E) for all peaks were identified. Peaks (*n* = 176 peaks) compared were putative peptide peaks based on mass defect (0.3–0.9 Da) and comparison to peptide databases from collagenase digestion. Almost all peptide data points were ≤10% peak intensity. All five breast sections were compared to one another to obtain the average relative percent change in peak intensities: A/B (0.93 ± 1.79%), A/C (0.34 ± 2.11%), A/D (1.45 ± 1.77%), A/E (1.85 ± 1.89%), B/C (−0.57 ± 2.49%), B/D (0.53 ± 1.72%), B/E (0.93 ± 2.12%), C/D (1.14 ± 2.25%), C/E (1.54 ± 2.44%), D/E (0.41 ± 2.18%). (**d**) Imaging mass spectra of peaks: 847.4532, 919.4466, 1019.5178, 1211.6325, 1242.6099, 1399.6745 *m*/*z*. (Mann–Whitney U test, *p*-value < 0.05). Breast cancer tissue samples *n* = 5.

An overall goal was to develop the approach towards understanding stromal heterogeneity in breast cancer risk and progression, therefore further studies focused on breast tissue. To understand the potential variation of peak intensity attributed to the heterogeneity among tissue serial sections, we first compared peak intensities of five nonstained breast tissue serial sections. All serial sections were simultaneously prepared for mass spectra acquisition using the workflow as described [35]. The same peak list was used for evaluation of reproducibility per consecutive tissue section (Table S1). Neighboring (~5 µm in distance) and distant slides (~30 µm in distance) labeled A-D were compared to each other to determine the relative percent change in peak intensity based on increasing distance between two tissue sections (Figure 2a). The distribution of relative standard deviations (RSD) from the relative peak intensities had a mean of 1.44 ± 0.79 (Figure 2b). The overall relative percent change for each peak intensity between neighboring and distant sections were determined (Figure S2) with the mean relative percent change from each pairwise serial section comparison varying by <5% (Figure 2c). Representative images of the imaging mass spectra show similar stromal patterns across all tissue sections (Figure 2d). Lastly, Spearman correlation analysis shows significant positive correlations between each pairing section with high rho (r_s_) values (r_s_ > 0.80, *p*-values < 0.0001) (Figure S3). Overall, MALDI-IMS analysis demonstrated limited variation of relative peak intensity and high reproducibility of technical replicates between nonstained breast tissue serial and distant sections.

**Figure 3:**
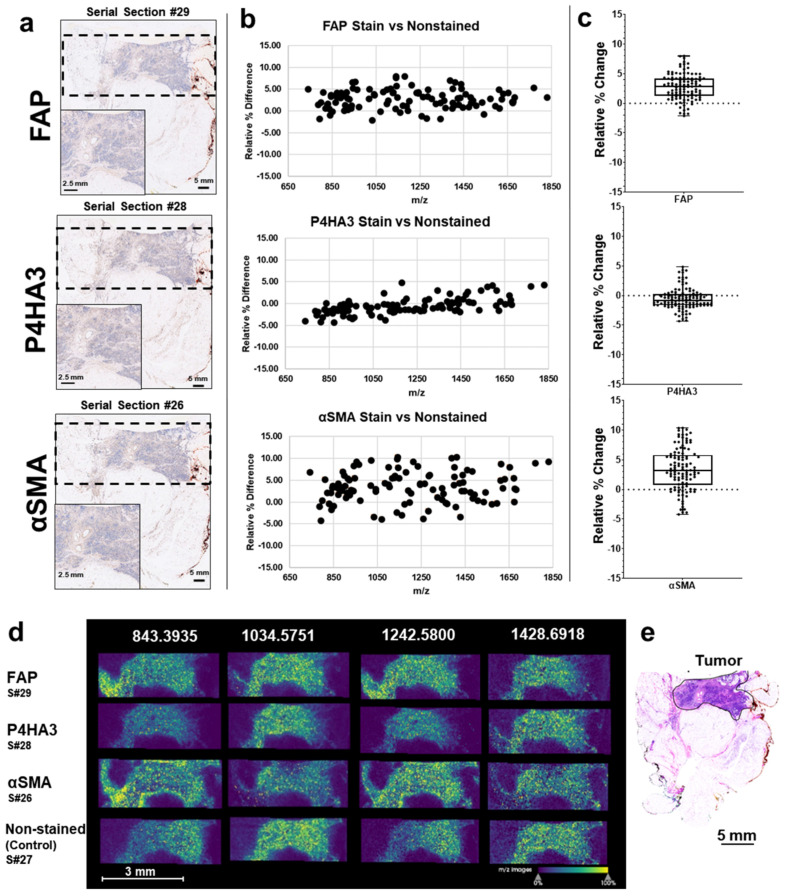
Imaging experiments after IHC stained (FAP, P4HA3, and αSMA) breast tissues show peak intensities are comparable with nonstained breast tissue. (**a**) Breast tissues were IHC stained with FAP, P4HA3, αSMA. Dashed line represents the regions that were imaged using MALDI IMS. (**b**) Relative peak intensity (111 peaks) differences between stained and nonstained tissue varied by −5% to +10%. (**c**) Mean relative percent change of stained compared to nonstained breast tissue. FAP, 2.78 ± 2.18%; P4HA3, −0.57 ± 3.18%; αSMA, 3.42 ± 3.54. (**d**) Representative imaging mass spectra of peaks from pre-stained and nonstained control tissue. Data are shown normalized to an internal standard. Step size was 150 × 150 µm by MALDI FT-ICR. (**e**) Tumor region annotated on an H&E stained breast tissue.

**Figure 4:**
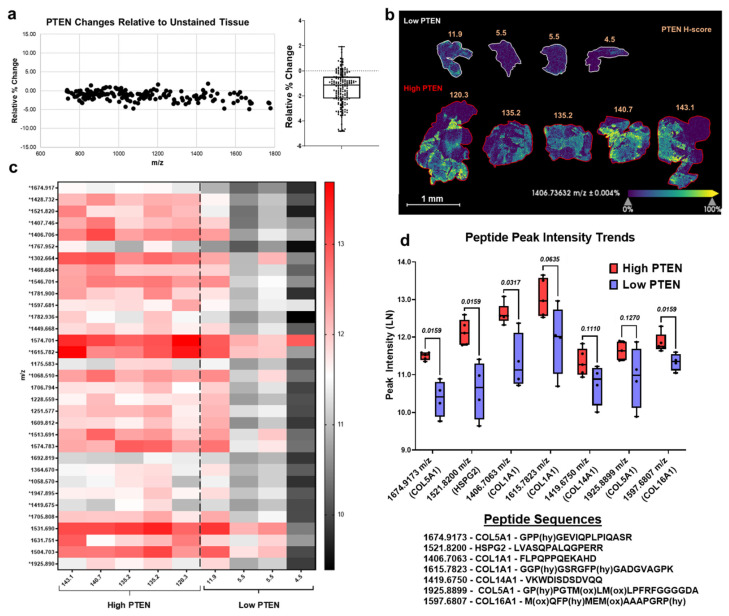
ECM peptides positively correlated with PTEN H-scores have a downward trend in Low PTEN tissues. (**a**) PTEN breast tissue staining showed comparable peak intensity (111 peaks) variance with nonstained breast tissue. Mean and interquartile range was determined for PTEN stained/nonstained breast tissues (−1.35 ± 1.30%). (**b**) Representative image of normal breast tissues scored by PTEN staining categorized as low and high PTEN from H-scores. (**c**) Heat map shows correlation of peaks (*m*/*z*) based on high and low PTEN categories. Peaks that were significantly correlated with PTEN H-score are annotated (*). (**d**) Putatively identified peptides that positively correlated with PTEN H-scores have lower peak intensities in low PTEN tissues relative to high PTEN tissues. (Mann–Whitney U test, *p*-value < 0.05). Low PTEN *n* = 3 biological replicates; high PTEN *n* = 4 biological replicates.

After observing relatively low peak intensity variability based on histology stains and breast serial sections, we performed IHC staining for breast tissue markers including fibroblast-activated protein (FAP), alpha smooth muscle actin (αSMA, activation of fibroblasts), prolyl 4-hydroxylase subunit alpha 3 (P4HA3, addition of hydroxylated proline to collagen) and phosphatase and tensin homologue deleted on chromosome 10 (PTEN, tumor suppressor protein) (Figure 3 and Figure S4a–c). Data from αSMA, P4HA3, and FAP stains demonstrate that when comparing each stain to a control nonstained tissue the mean relative percent change was <8% for all stains (Figure 3a). Additionally, Spearman correlation analysis demonstrates high correlation between stained and nonstained tissue with values of r_s_ ≥ 0.98 (Figure S5). PTEN staining followed by stromal-targeting IMS, as depicted in the schematic workflow (Figure 1), resulted in peak intensity variation of <3.5% when compared to nonstained control breast tissue (Figure 4a). Overall, histology-directed IMS allows for targeted stromal analysis on the same tissue section with comparable stromal peptide intensities as seen in nonstained tissue sections.

### 3.4. Proteomics Demonstrate Heterogeneous PTEN Expression on Tissue Regulate Extracellular Matrix (ECM) Proteins

To understand the influence that PTEN staining patterns may have on normal breast stroma, the developed workflow (Figure 1) was used to qualitatively analyze ECM peptide regulation. In normal breast tissue, histology scores (H-scores) of PTEN staining were used in order to analyze the relationship between low and high categorized PTEN staining expression and ECM proteins. PTEN H-scores were previously calculated based on cell staining intensity and number of cells stained as described [37]. Tissues were categorized as high PTEN (H-scores 120.3–143.1) or low PTEN (H-scores 4.5–11.9) (Figure S6). A representative peak (1406.7363 *m*/*z*) shows heterogeneous peak expression and distinct distributions between low and high PTEN tissues (Figure 4a). Low PTEN tissue demonstrated lower peak intensity patterns compared to high PTEN (*p* < 0.05) (Figure 4b). Of the peaks that positively correlated, 21 out of 33 peaks were found to be significantly correlated with PTEN H-scores (*p* < 0.05) (Figure 4c). An exception was one low PTEN tissue with an H-score of 11.9 which showed peak expression patterns that more closely resembled the high PTEN group patterns. This tissue was observed to show overall low PTEN but had a small number of intensely staining PTEN regions. High and low PTEN tissues showed relative distinct peptide peak expression treads when comparing the two groups with low PTEN samples having downward trends (Figure 4d). To further identify the specific ECM proteins and their associated post-translational modifications that may be regulated by PTEN, high-resolution accurate mass (HRAM) LC-MS/MS proteomics was conducted for high and low PTEN tissue profiles. Peptides from the same tissues were compared by accurate mass back to the high mass accuracy imaging data. Peptides belonging to COL5A1, COL1A1, COL14A1, COL16A1 and HSPG2 proteins showed downregulated peptide peak intensities in low PTEN compared to high PTEN tissue (Figure 4d, Table 1). Overall, PTEN H-scores appear to be associated with distinct peptide peak intensity patterns with specific peptides trending in the same direction as PTEN scores.

**Figure 5:**
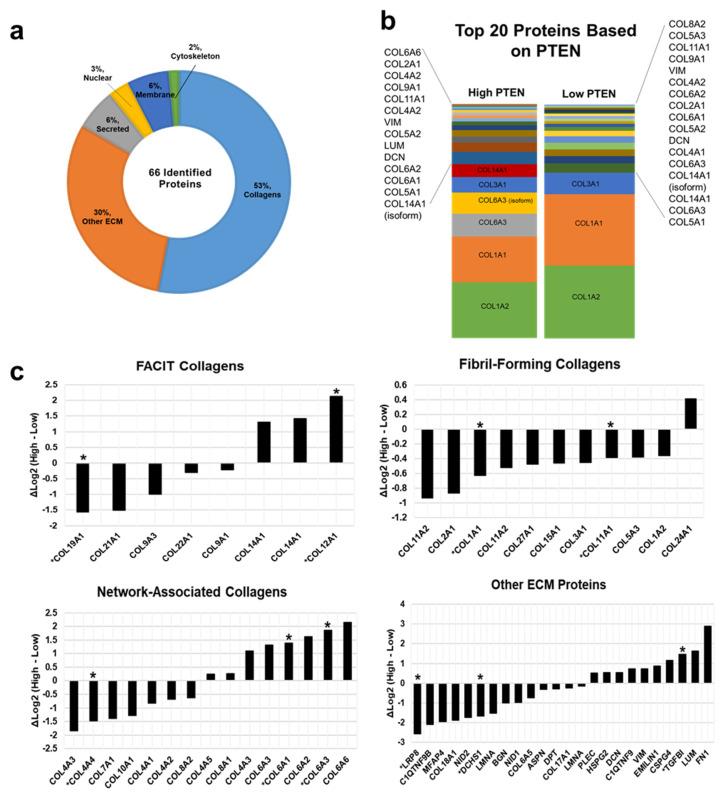
Identified proteins from LC/MS proteomic analysis demonstrate PTEN regulated expression patterns of ECM proteins in breast tissue. (**a**) Sixty-six proteins identified and ranked based on relative intensity. The proteins identified were collagens (53%), ECM (30%), secreted (6%), nuclear (3%), membrane (6%) and cytoskeleton proteins (2%). (**b**) Proteins ranked by peak intensity based on PTEN expression (high and low PTEN). Top 20 ranked proteins expressed were identified and relatively compared. (**c**) Negative ΔLog2 represent protein upregulation in low PTEN and positive ΔLog2 represent protein upregulation in high PTEN. Proteins that were statistically significantly regulated (two-tailed student *t*-test *p*-value < 0.05) are annotated (*) such as COL19A1, COL12A1, COL1A1, COL11A1, COL4A4, COL6A3, COL6A1, LRP8, DCHS1 and TGFB1.

Overall, the HRAM proteomic analysis revealed 66 putatively identified proteins from breast tissue that were classified as collagen (53%), ECM (30%), secreted (6%), membrane (6%), nuclear (3%) and cytoskeletal (2%) proteins (Figure 5a). The proteins were ranked based on intensity (Ln) and then the top 20 proteins from each category (high PTEN and low PTEN) were determined and displayed based on relative abundance (Figure 5b). Log fold change of relative protein abundance was calculated and graphed based on protein classification: fibril-forming collagens, fibril-associated collagens with interrupted triple helices (FACIT) collagens, network-associated collagens and noncollagen proteins (Figure 5c). A negative fold change represents protein upregulation in low PTEN while a positive fold change represents protein upregulation in high PTEN. Of the total 66 proteins, 10 were found to be differentially regulated based on PTEN expression (Figure 5c). Proteins COL1A1, COL11A1, COL4A4, COL19A1, DCHS1 and LRP8 were significantly upregulated in low PTEN tissue profiles (*p* < 0.05) (Figure 5c). Proteins COL6A3, COL6A1, COL12A1 and TGFB1 were significantly upregulated in high PTEN tissue profiles (*p* < 0.05) (Figure 5c). To conclude, HRAM proteomics suggested that certain ECM proteins, and in particular specific collagen types, may be differentially regulated based on PTEN expression patterns in normal breast tissue.

### 3.5. PTEN Stained Breast Cancer Tissue Microarrays Reveal Distinct Peptide Peak Expression Patterns Based on Staining and Tumor Region

**Figure 6:**
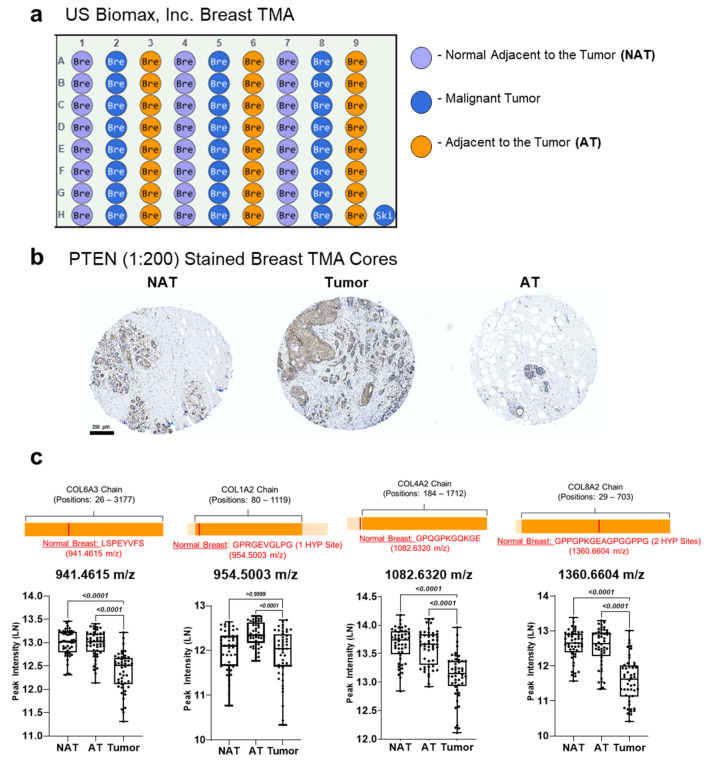
PTEN-stained human breast TMA datasets demonstrate unique peptide peak intensity patterns based on distinct tumor regions. (**a**) Outline of breast tumor microarrays (TMAs). (**b**) Representative images of PTEN (1:200) stained breast TMA cores. (**c**) Illustration of estimated location of putatively identified peptide sequence on collagen chain using a normal breast tissue proteomic database. LN transformed peak intensity data are demonstrated via scatter plots. Two-tailed student *t*-test (*p*-value < 0.05) was used to compare mean intensity of specific peptide peaks. HYP = hydroxylated proline. (Mann–Whitney U test, *p*-value < 0.05). Breast TMAs include 134 cases and 144 cores.

Application of the histology-directed IMS was done on human breast TMA cores characterized as normal adjacent to the tumor (NAT), adenosis adjacent to tumor (AT) and malignant tumor (Tumor) (Figure 6a). Autostained PTEN demonstrated heterogeneous tissue patterns among tumor, AT and NAT cores (Figure 6b and Figure S7). IMS analysis revealed specific peaks that significantly differed based on TMA core type (Figure 6c). Peaks were assigned putative identifications based on the described proteomics done on normal breast with high/low PTEN patterns. Collagen peptides demonstrated differential peak intensities between the three groups with tumor cores consistently having significantly lower peak intensities compared to AT cores and most of NAT cores (Figure 6c). Comparison of PTEN staining from NAT cores demonstrated significant positive correlation with only one peak, while tumor cores positively correlated to eight peaks (Figure S8a,c). PTEN staining area of AT cores showed significant negative correlation with four peptide peaks (Figure S8b). Lastly, receiver operating characteristic (ROC) curve analysis revealed significant area under the curve (AUC) values (AUC ≥ 0.70, *p* < 0.05) for unique peaks (Table 2 and Figure S9). Overall, histology-directed IMS analysis of PTEN stained TMA cores further suggests differential collagen peptide regulation that may be influenced by PTEN and tumor regions in breast tissue.

## 4. Discussion

Understanding the heterogeneity of the tumor microenvironment is essential towards more targeted cancer therapeutics and better prognostic tools. Previous studies have focused on hallmarks of cancer such as the ability of cancer cells to sustain proliferative signaling, evade growth suppressors and activate invasion and metastasis [49,50]; however, the tumor-associated stroma has also been shown to have critical implications in tumorigenesis, invasion, and metastasis [5,51,52,53]. The current study shows the feasibility of linking the identification of cellular heterogeneity based on IHC staining patterns and to their associated ECM regulation within a tumor microenvironment.

Within the breast cancer stroma, the connective tissue components are all involved in orchestrated interactions important for regulation of cell survival, proliferation and cell death. In cancers such as breast carcinomas, cancer-associated fibroblasts (CAFs) are the most prominent stromal cell type [54]; CAFs have been shown to significantly deposit and regulate stromal components such as collagen proteins, which have the potential to promote tumor progression and therapeutic resistance [52,55,56]. However, less is known about the influence that specific fibroblasts have on altering the dysregulated stroma composition and signaling seen in cancer. However, a significant challenge is studying the stromal components of the complex tumor microenvironment that may be influenced by cellular components with unique staining patterns.

To connect imaging strategies with cell specific signaling, we adapted an approach that integrates immunohistochemistry with mass spectrometry studies on the same tissue section for better understanding of fibroblast specific control of the ECM. Matrix-assisted laser desorption/ionization imaging mass spectrometry (MALDI IMS) can be utilized to generate both spatial and quantitative analysis of the vast variety of ECM proteins found on tissue [34,35,36,45,46]. In addition to obtaining map localization of proteins with respect to specific cells and the tumor, this imaging modality can also report putatively identified post-translational modifications of these proteins [35,36,43,45,46,47]. The developed workflow used in this study was aimed to investigate proteomic acquired stromal analysis of formerly immunohistochemistry stained breast and hepatocellular carcinoma tissue with markers FAP, αSMA, P4HA3 and PTEN.

In a proof-of-concept experiment, we tested the aforementioned approach of integrating IHC and imaging using PTEN-stained tissues from normal breast. PTEN is a negative regulator of the phosphoinositide 3-kinase (PI3K) pathway and downregulation of PTEN is a common feature of activated tumor-associated stroma [15,18,20]. PTEN depletion has been found to promote collagen alignment and remodeling of collagen at the tumor edge [15,18]. The ECM in the tumor stroma constantly undergoes structural changes characterized by collagen degrading, redepositing, cross-linking and stiffening during cancer invasion [3,13,14,57,58,59,60]. More specifically, collagens types I, II, VI, and XI, have been associated with poor prognosis and tumorigenesis in different cancers [58]. The specific collagen subtypes and their protein regulation are highly dependent on the tissue type, fibroblast subtype and the ECM microenvironment. Thus, observing differential collagens associated with high PTEN (tumor-suppressing) or low PTEN (tumor-promoting) suggests that fibroblast subtypes designated by PTEN status express unique collagen proteomes. Currently, classification of “tumor-promoting” and “tumor-suppressing” collagen subtypes is an area that demands more research. Here, we show that ECM proteins including collagen subtypes (fibril-forming, network-associated and FACIT) and other ECM proteins are differentially expressed in breast tissue based on high and low PTEN expression patterns.

To further investigate PTEN staining expression patterns and their relation to stromal proteins, PTEN H-scores were used to evaluate peptide peak intensity expression. Based on our findings, there is differential peptide peak intensities based on PTEN suggesting a potential role for PTEN in regulating stromal proteins. While we classified PTEN stained breast tissue as “low” or “high”, it is important to note that these binary terms represent the averaged stained score for the whole tissue, which may limit information regarding localized “hot spots” of higher PTEN intensity expression in specific areas. For example, a breast tissue sample with a PTEN H-score of 11.9 categorized as low PTEN demonstrated highly localized similar peak intensities as those categorized as high PTEN, suggesting diverse PTEN staining expression patterns on the tissue. Lastly, human breast TMAs were used to study the relationship between PTEN expression patterns and peptide peak intensities based on different tumor regions (NAT, AT and tumor). Our findings illustrate distinctive PTEN staining patterns based on tumor region, emphasizing the heterogeneity seen within the tumor microenvironment. Additionally, PTEN staining area was shown to significantly correlate with unique collagen peptide peaks suggesting differential collagen peptide regulation may be influenced by PTEN as well as tumor region. This gives rise to future considerations for PTEN stromal analysis such as focusing on evaluating tissues based on sections to account for PTEN intensity variability per area of tissue.

With breast cancer tissue being highly diverse, techniques with the potential to capture the impact of complex stroma, especially the regulation of the extracellular matrix, are critical for understanding the stroma’s roles in breast cancer risk and progression. Different staining techniques such as H&E and immunohistochemistry (IHC) have been advantageous for characterizing breast histology and histopathology; meanwhile, mass spectrometry represents a useful translational tool for proteomic analysis. In the current study, we report the combination of chromophoric immunohistochemistry to define cell types and their ECM niches. Additional methods may be easily adapted to this workflow. For instance, in histology-directed MALDI mass spectrometry, a tissue section is stained with H&E and then annotated for specific cells of interest [37]. On a serial section, the laser is then guided to target the specific cell types using the annotations from the serial stained section and pixel coordinates for accuracy. Here, the identical IHC stained section could be used for targeting not only specific cell types, but their surrounding niche. Combinations of multiplexed IHC with targeted proteomic access could greatly expand our knowledge of cell subtypes in cancer [61]. Current quantitative approaches in imaging mass spectrometry include on-tissue spotting of standards, internal standards and the mimetic models [62,63,64,65,66]. An additional advancement to this method could be made by utilizing synthesized peptide standards of the ECM components to obtain quantitative information. Overall, integrating immunohistochemistry of cell markers with imaging mass spectrometry is a growing, yet challenging, field that can greatly expand on proteomic studies in relation to cancer risk and progression.

## 5. Conclusions

In conclusion, our study demonstrates that acquisition of ECM data by targeted imaging mass spectrometry after IHC staining results in comparable changes in peak detection relative to replicate analysis of nonstained controls. Our developed method can generate qualitative and relatively quantitative proteomics analysis of ECM peptides that may be potentially regulated by stromal markers such as PTEN. The approach will also be useful in understanding other cell types such as immune cells and their ECM program within the tumor microenvironment. Overall, this study increases the clinical significance of ECM stromal proteins found within the tissue microenvironment. The combined data will be useful towards understanding patient specific responses to therapies and for prognostic or diagnostic stratification.

## Figures and Tables

**Table 1:** ECM peptides with corresponding *m*/*z*, sequence, amino acid (AA) positon and Spearman r value. In parentheses is the probability of the post translational modification on the corresponding amino acid residue. (P^X^ = hydroxylation of proline). The *p*-value for Spearman’s correlation was ≤0.005. * Putative identification. Sequences with hydroxyproline site probabilities in parenthesis ^a^, Proline site modification ^b^, and Peptide score (−log probability) ^c^.

Gene Name	Sequence ^a^	Modified ^b^	AA Position	Peptide Score ^c^	Theoretical M + H	Observed *m*/*z*	PPM	H-Score Correlation
COL5A1	GP^x^(0.015)P^x^(0.985)GEVIQPLPIQASR	1568; 1569	1567–1582	321.03	1674.9173	1674.9173	0.03	0.92
*HSPG2	LVASQPALQGPERR	No	1444–1457	104.41	1521.8496	1521.8200	*19.5	0.87
COL1A1	FLPQPPQEKAHD	No	1200–1211	80.98	1406.7063	1406.7063	0	0.83
COL1A1	GGP^x^(1)GSRGFP^x^(1)GADGVAGPK	490; 496	488–505	62.20	1615.7823	1615.7823	0	0.73
COL14A1	VKWDISDSDVQQ	No	753–764	95.57	1419.6750	1419.6750	0.04	0.73
COL5A1	GP^x^(0.5)P^x^(0.5)GTMLMLPFRFGGGGDA	515; 516	514–532	75.09	1925.8884	1925.8899	−0.76	0.73
COL16A1	MQFP^x^(1)MEMAAAP^x^(0.436)GRP^x^(0.564)	1465; 1472; 1475	1462–1475	75.29	1597.6807	1597.6807	0.03	0.71

**Table 2:** Comparison of breast cancer TMAs from malignant tumor, normal adjacent to the tumor (NAT), and adjacent to the tumor (AT) using area under the receiver operating curve (AUC) ≥0.70. (*p*-value < 0.0001 indicated by *).

Peak *m*/*z*	NAT vs. Tumor	AT vs. Tumor	NAT vs. AT
941.4615	0.90 *	0.88 *	0.52
954.5003	0.54	0.78 *	0.74 *
1082.632	0.87 *	0.84 *	0.56
1360.6604	0.90 *	0.86 *	0.51

## Data Availability

Data are contained within the article or supplementary material. The mass spectrometry proteomics data have been deposited to the ProteomeXchange Consortium (http://proteomecentral.proteomexchange.org, publicly accessible: 31 August 2022) via the PRIDE partner repository [67] with the dataset identifier PXD028107.

## Supplementary Materials

The following are available online at https://www.mdpi.com/article/10.3390/cancers13174419/s1, Figure S1. Stained hepatocellular carcinoma sections with H&E, nuclear stain, trichrome, SOD2 and glypican demonstrate relatively low peak intensity variability compared to nonstained tissue, Figure S2. Scatter plots of serial breast sections show low variation in peak intensity in consecutive tissue sections of unstained breast tissue, Figure S3. Serial unstained breast sections demonstrate reproducibility of technical replicates using consecutive tissue sections, Figure S4. αSMA-, FAP- and P4HA3-stained breast cancer tissues, Figure S5. IHC-stained breast sections with αSMA, FAP, and P4HA3 demonstrate high correlation with unstained tissue, Figure S6. Normal breast tissues were stained with PTEN and scored, Figure S7. PTEN-stained human breast TMAs, Figure S8. PTEN-stained human breast TMA datasets demonstrate significant correlation between PTEN staining area and peak intensity, Figure S9. ROC analysis of PTEN-stained human breast TMA datasets, Table S1. List of peaks used for evaluation of reproducibility per consecutive tissue section.
