# Supplementary material for: Defining the Tumor Microenvironment by Integration of Immunohistochemistry and Extracellular Matrix Targeted Imaging Mass Spectrometry
Source: Cancers (Basel). 2021 Sep 1;13(17):4419. doi: 10.3390/cancers13174419 (PMC8430776; doi:10.3390/cancers13174419)
Supplement: Supplementary file 1 [file cancers-13-04419-s001.zip › cancers-1304067-supplementary.pdf]

cancers

MDPI

Supplementary Material: Defining the Tumor Microenvironment by Integration of Immunohistochemistry and Extracellular Matrix Targeted Imaging Mass Spectrometry

Denys Rujchanarong, Julia Lefler, Janet E. Saunders, Sarah Pippin, Laura Spruill, Jennifer R. Bethard, Lauren E. Ball, Anand S. Mehta, Richard R. Drake, Michael C. Ostrowski and Peggi M. Angel

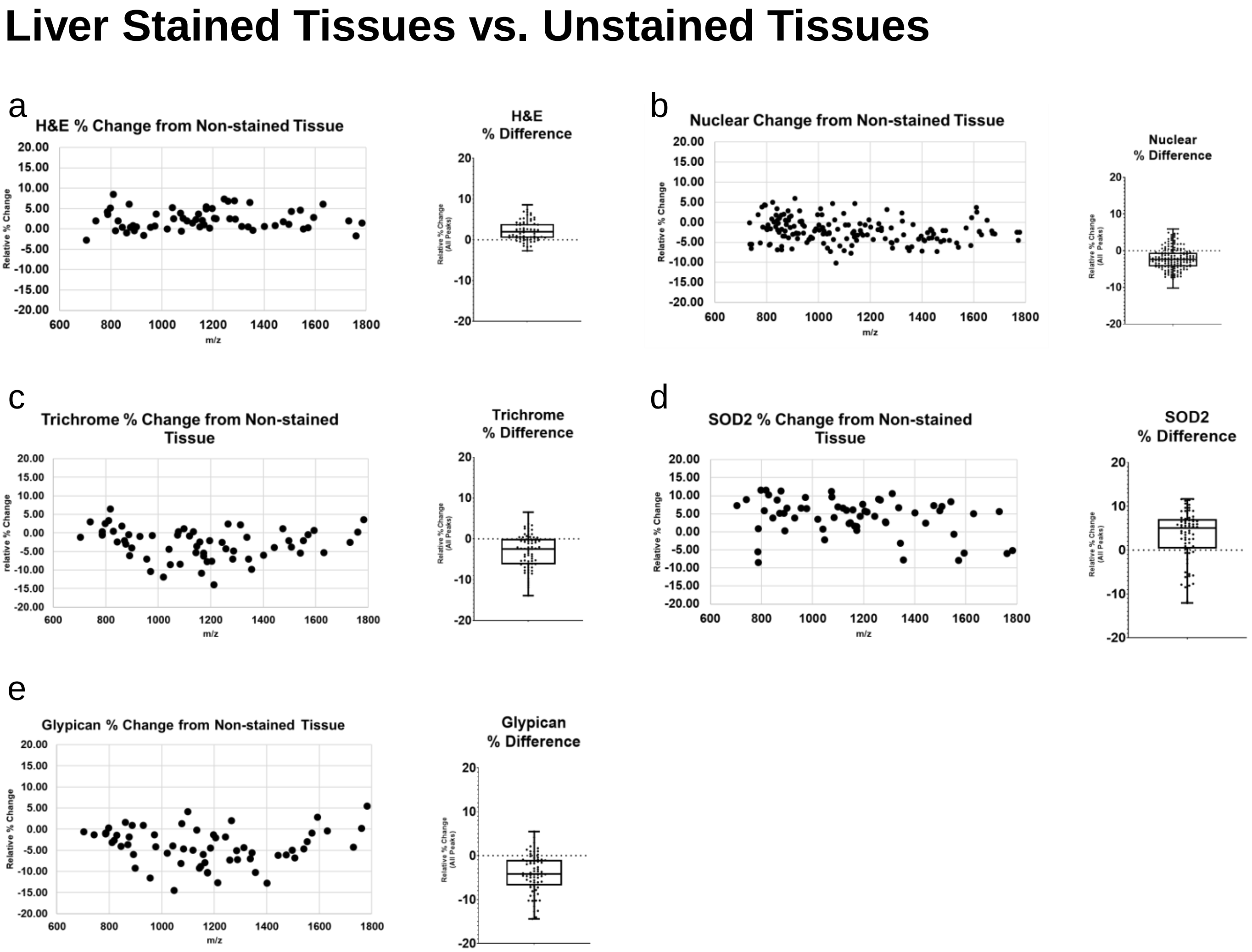

Figure S1. Stained hepatocellular carcinoma sections with H&E, nuclear stain, trichrome, SOD2 and glypican demonstrate relatively low peak intensity variability compared to non-stained tissue. LN transformed peak intensity data are demonstrated via scatter plots. The relative mean intensities compared to non-stained control tissue were calculated for (**a**) H&E: (2.22 ± 2.46%), (**b**) nuclear (−2.15 ± 2.97%), (**c**) trichrome (−3.22 ± 4.23%), (**d**) SOD2 (3.22 ± 5.87%), and (**e**) glypican staining (−4.15 ± 4.34%).

Breast Variability

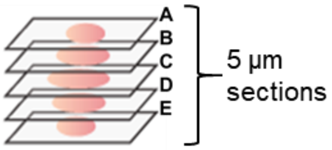

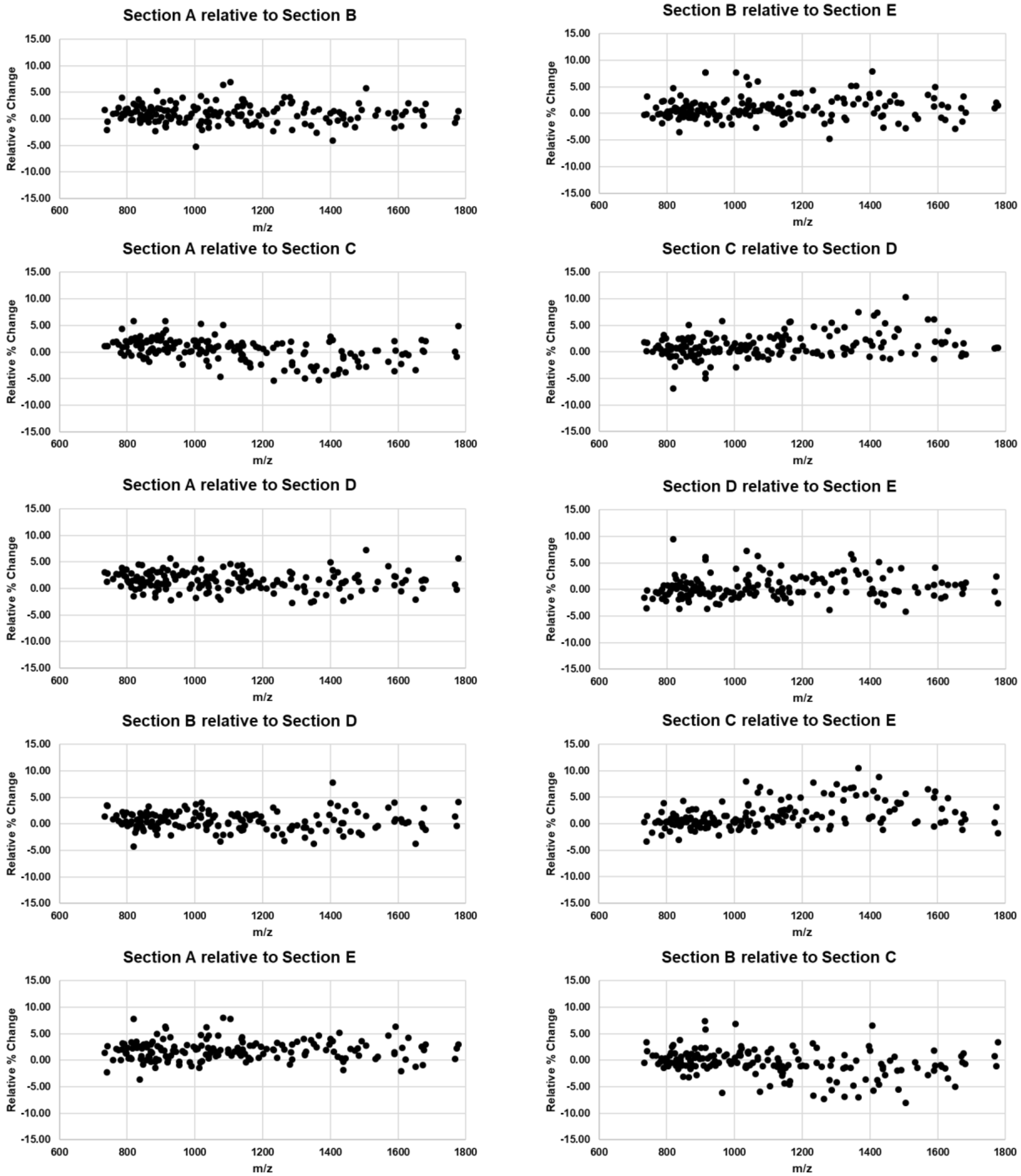

Figure S2. Scatter plots of serial breast sections show low variation in peak intensity in consecutive tissue sections of unstained breast tissue. Peak intensity ratios from non-stained tissue were compared to determine expected variability in consecutive tissue sections. Plots are ratios of same peak intensities from section X to section Y over 600-1800 m/z. Each data point represents a peak. Peak comparison show majority of peak intensities are within 5% of serial sections and within 10% of each other from sections that are further apart (distant).

Breast Variability

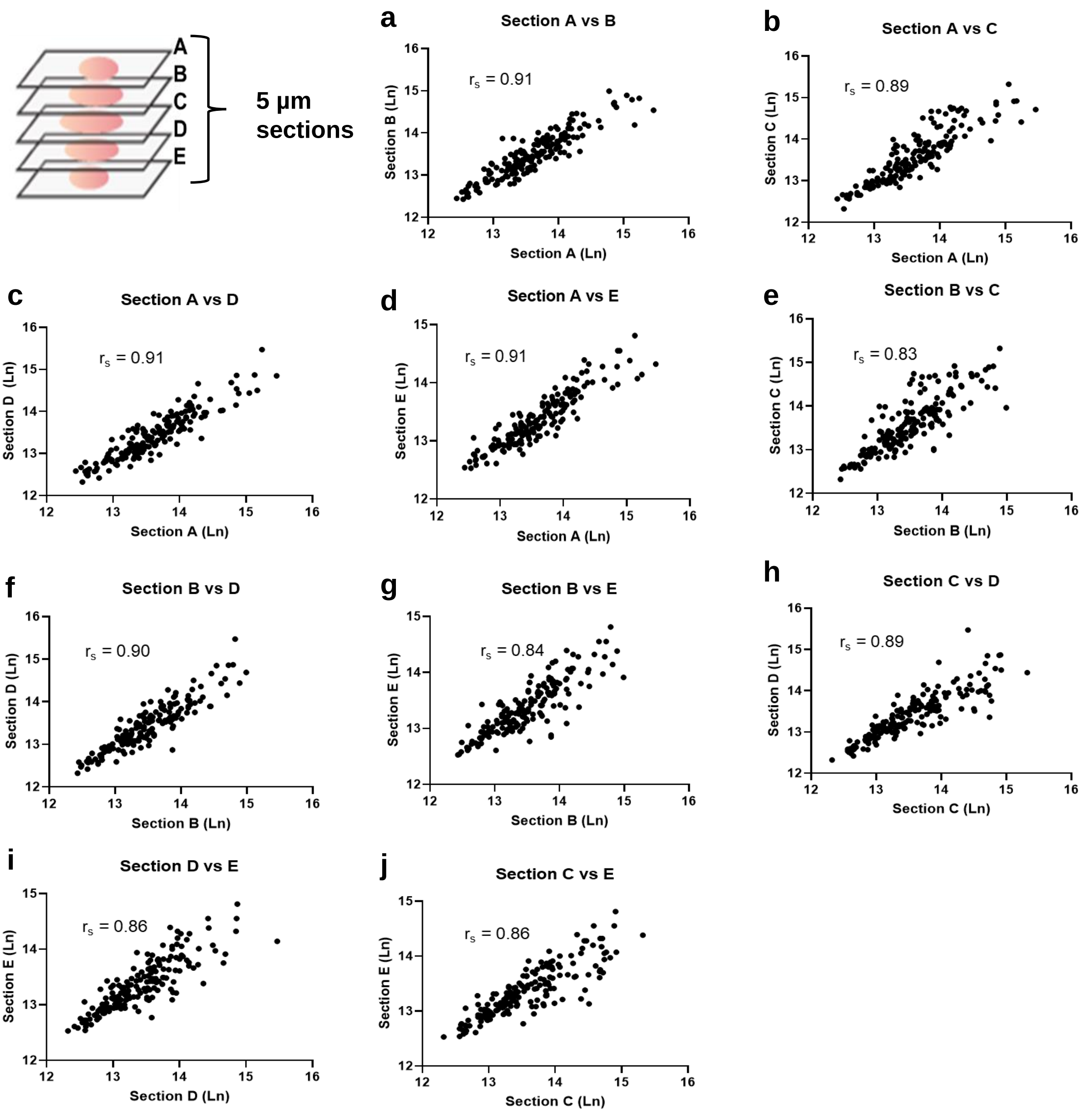

Figure S3. Serial unstained breast sections demonstrate reproducibility of technical replicates using consecutive tissue sections. Spearman's rank-order correlation coefficient r_s was used as a secondary test to evaluate correlation between consecutive tissue sections. Peak range was 600-1800 m/z. Each point represents a peak. All correlation plots generated a *p*-value <0.0001 (alpha = 0.05).

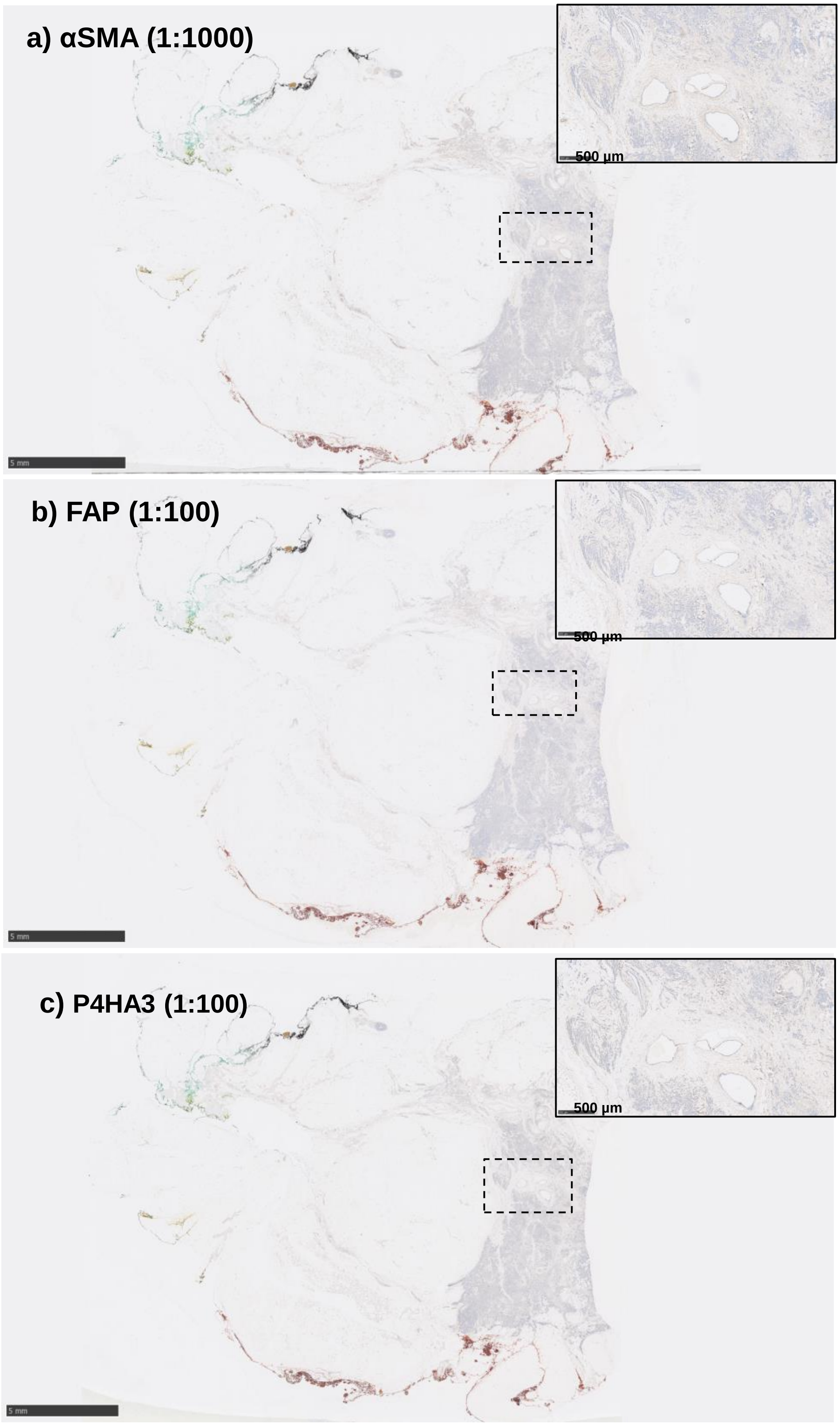

Figure S4. αSMA, FAP and P4HA3 stained breast cancer tissues. (**a**) αSMA was stained overnight using 1:1000 dilution. (**b**) FAP was stained overnight using 1:100 dilution. (**c**) P4HA3 was stained overnight using 1:100 dilution

IHC Stain Variability: Stained relative to Non-stained

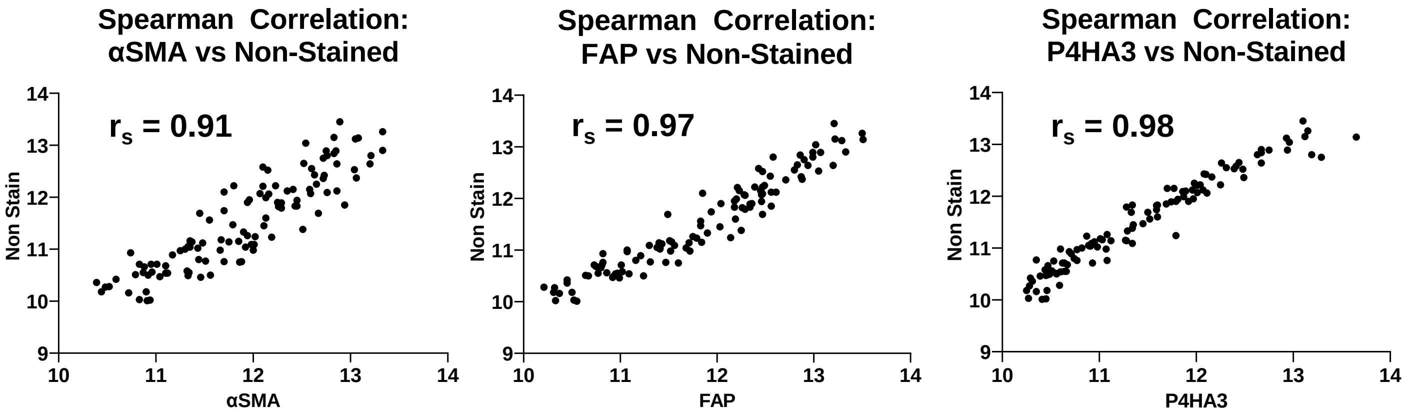

Figure S5. IHC stained breast sections with *α*SMA, FAP, and P4HA3 demonstrate high correlation with unstained tissue. Spearman's rank-order correlation coefficient (r_s) was used to evaluate correlation between tissue sections. Peak range was 650-1850 m/z. Each point represents a peak. All correlation plots generated a *p*-value <0.0001 (alpha =0.05).

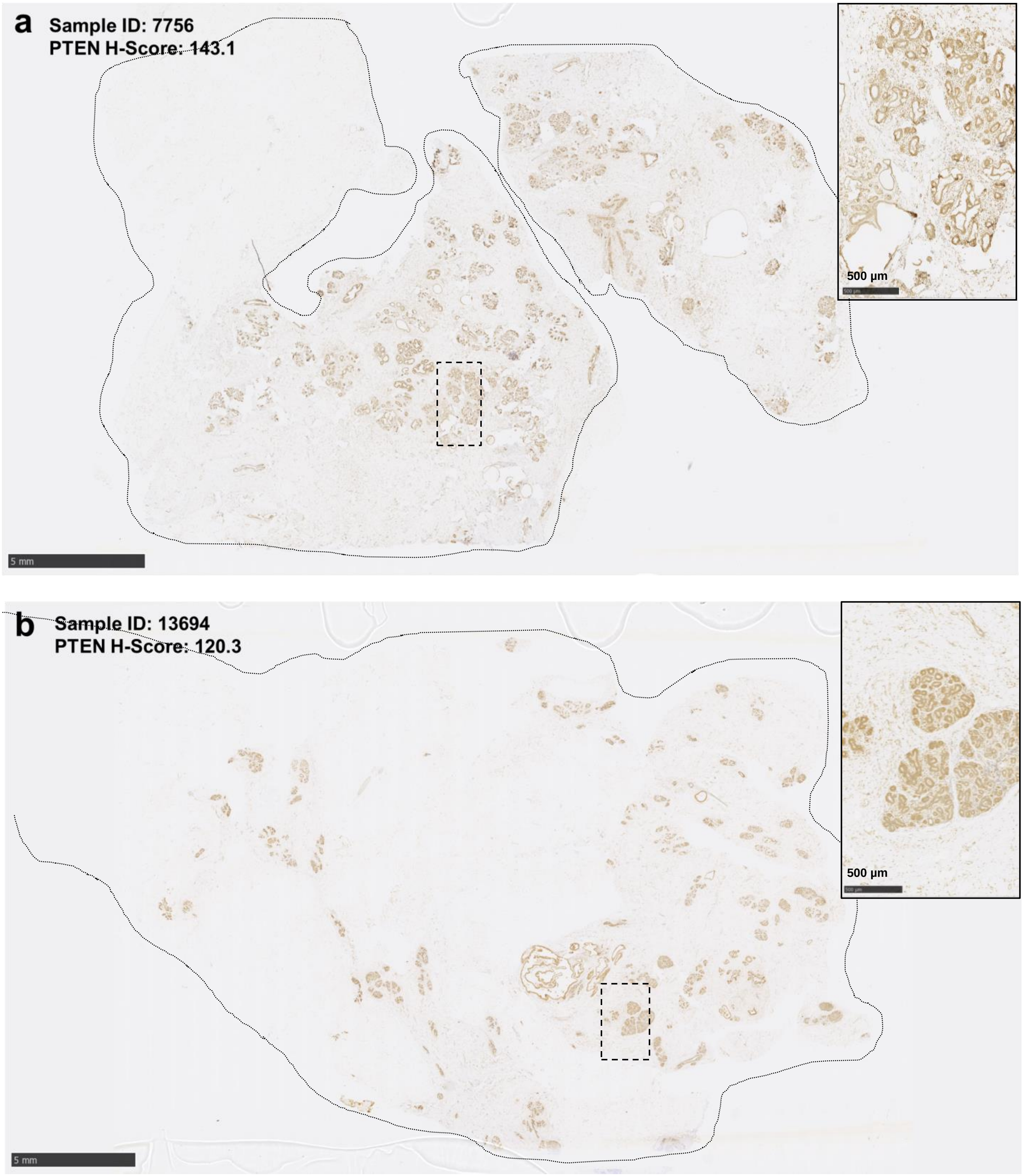
a
Sample ID: 7756
PTEN H-Score: 143.1
500 µm
5 mm
b
Sample ID: 13694
PTEN H-Score: 120.3
500 µm
5 mm

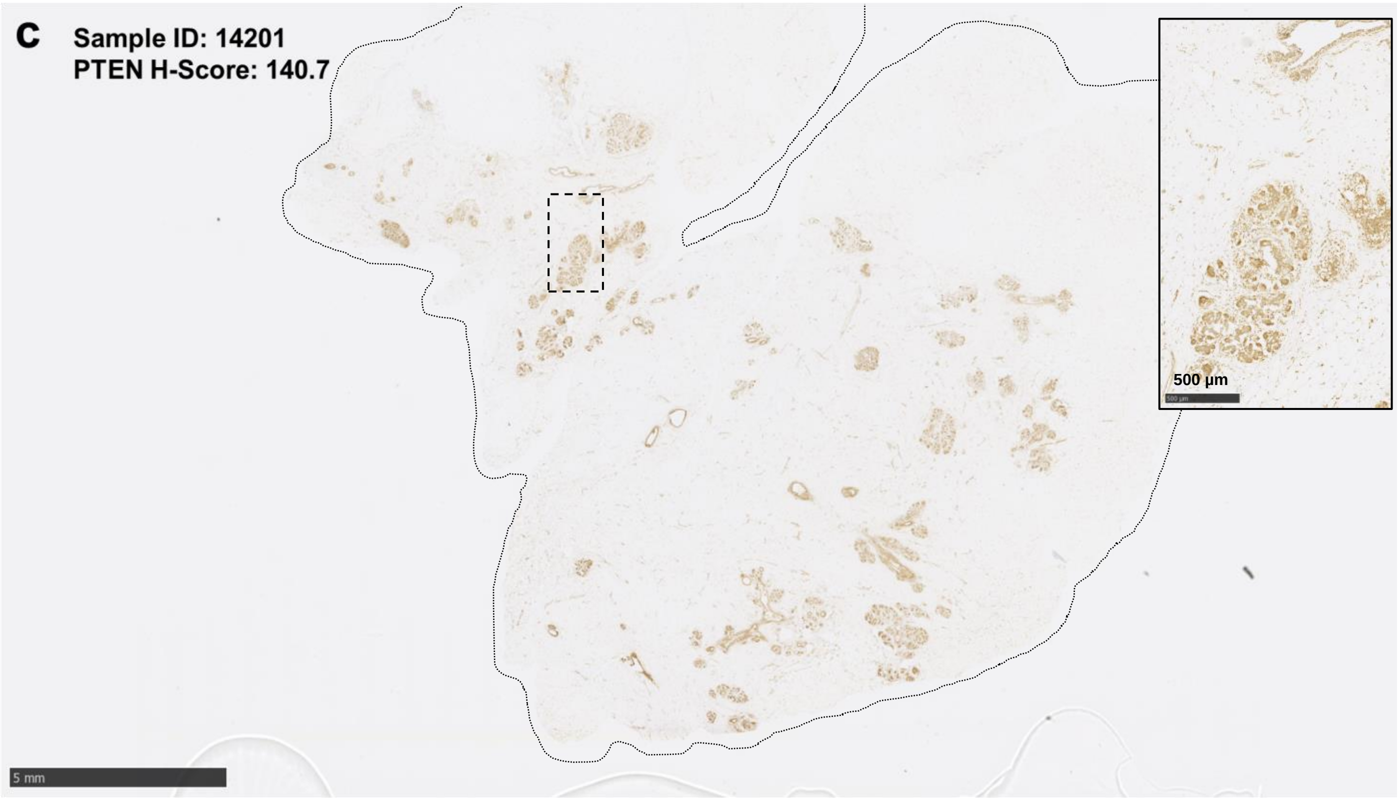
c
Sample ID: 14201
PTEN H-Score: 140.7
500 µm
5 mm

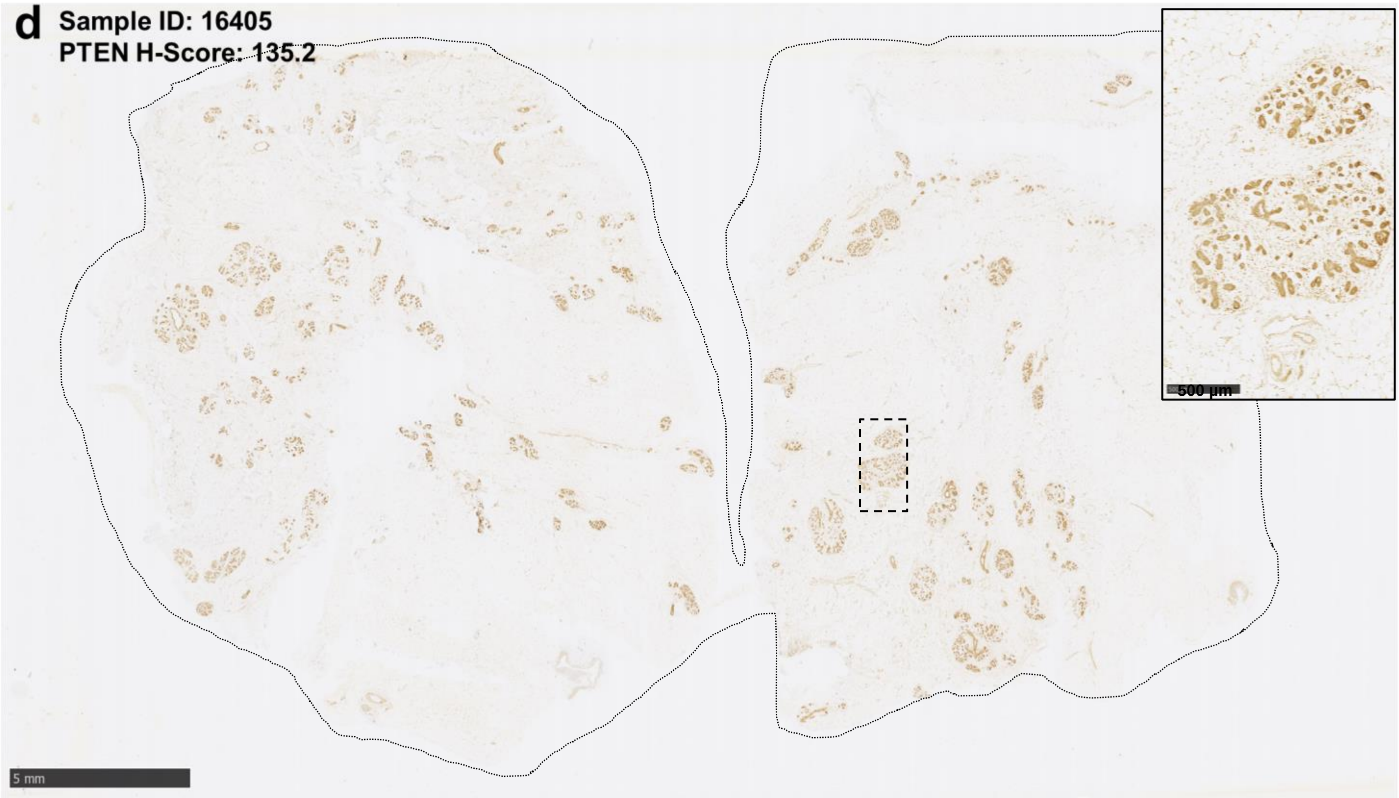
d
Sample ID: 16405
PTEN H-Score: 135.2
500 µm
5 mm

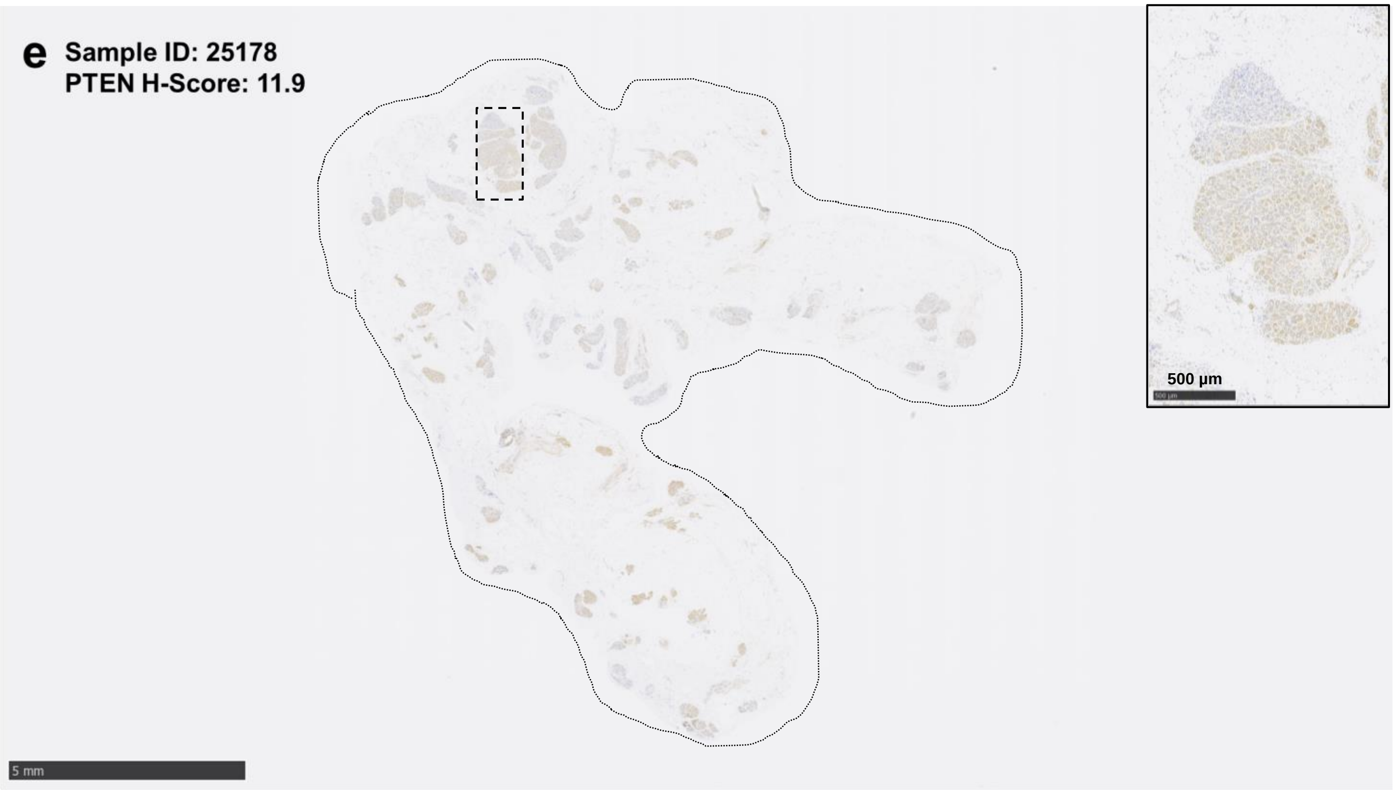
e
Sample ID: 25178
PTEN H-Score: 11.9
500 µm
5 mm

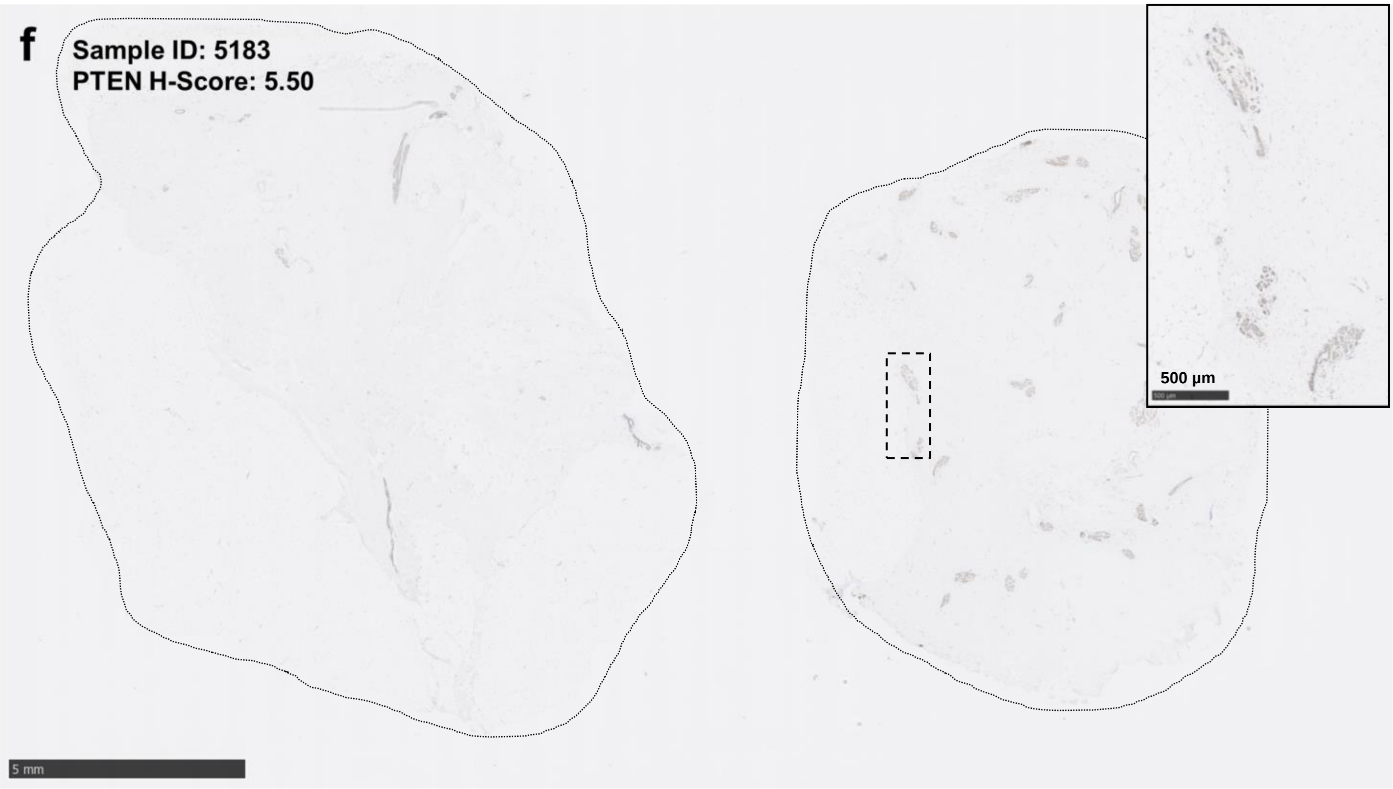
f
Sample ID: 5183
PTEN H-Score: 5.50
500 µm
5 mm

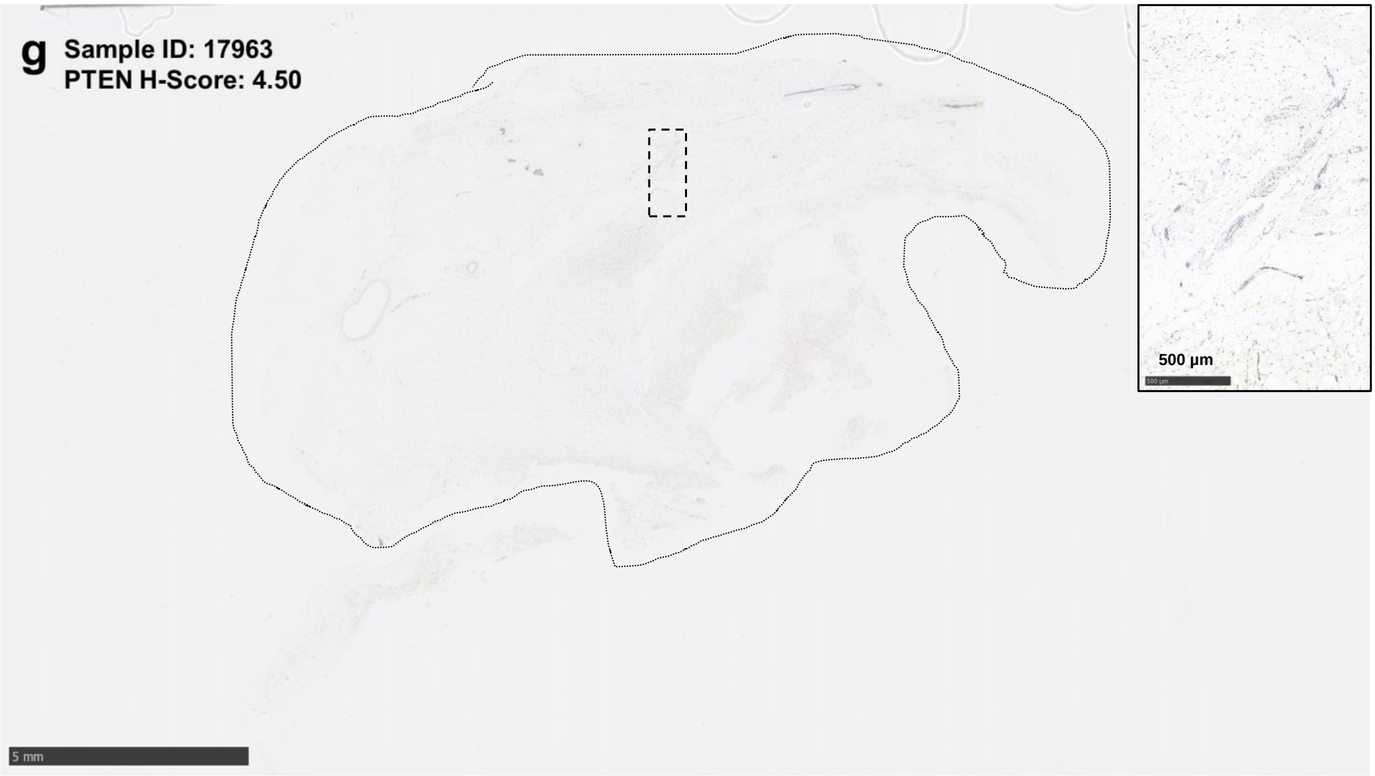

Figure S6. Normal breast tissues were stained with PTEN and scored. Normal breast tissue were categorized as either High PTEN (a–d) or Low PTEN (e–g) based on representative PTEN immunohistochemistry and quantification based on relative intensity and number of cells stained (Sizemore et al, Nature Comm 9:2783).

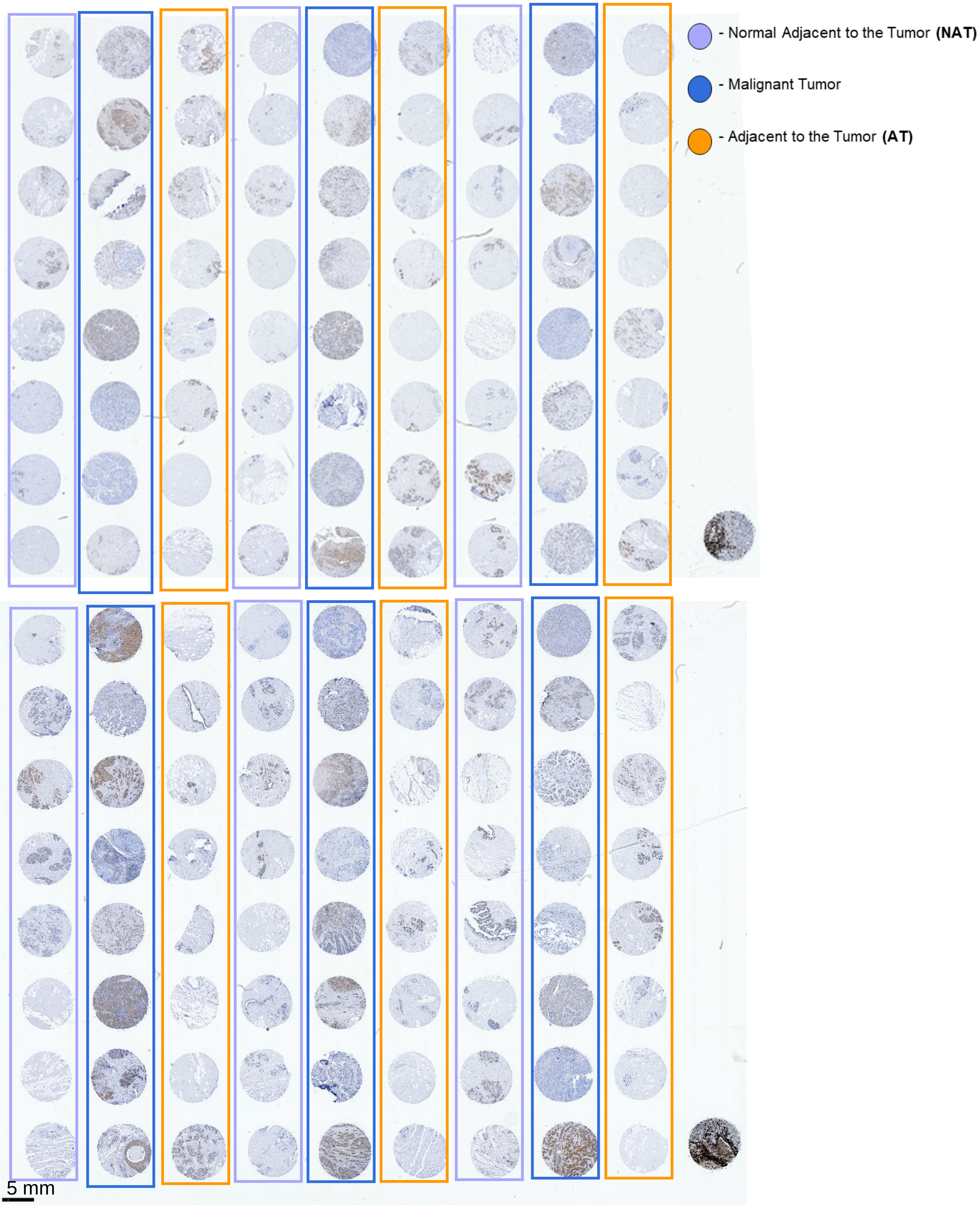

Figure S7. PTEN stained human breast TMAs. PTEN (1:200).

a Normal Adjacent to Tumor Correlation Plots

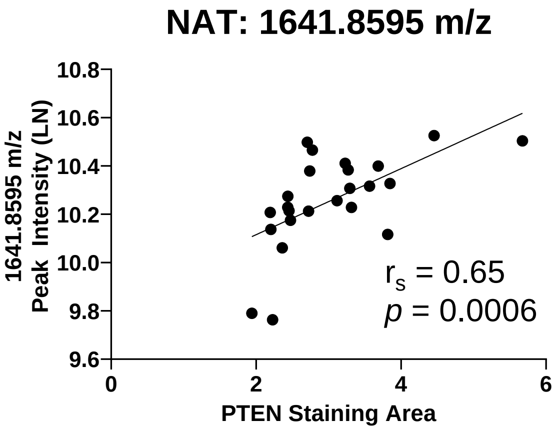

b Adjacent to Tumor Correlation Plots

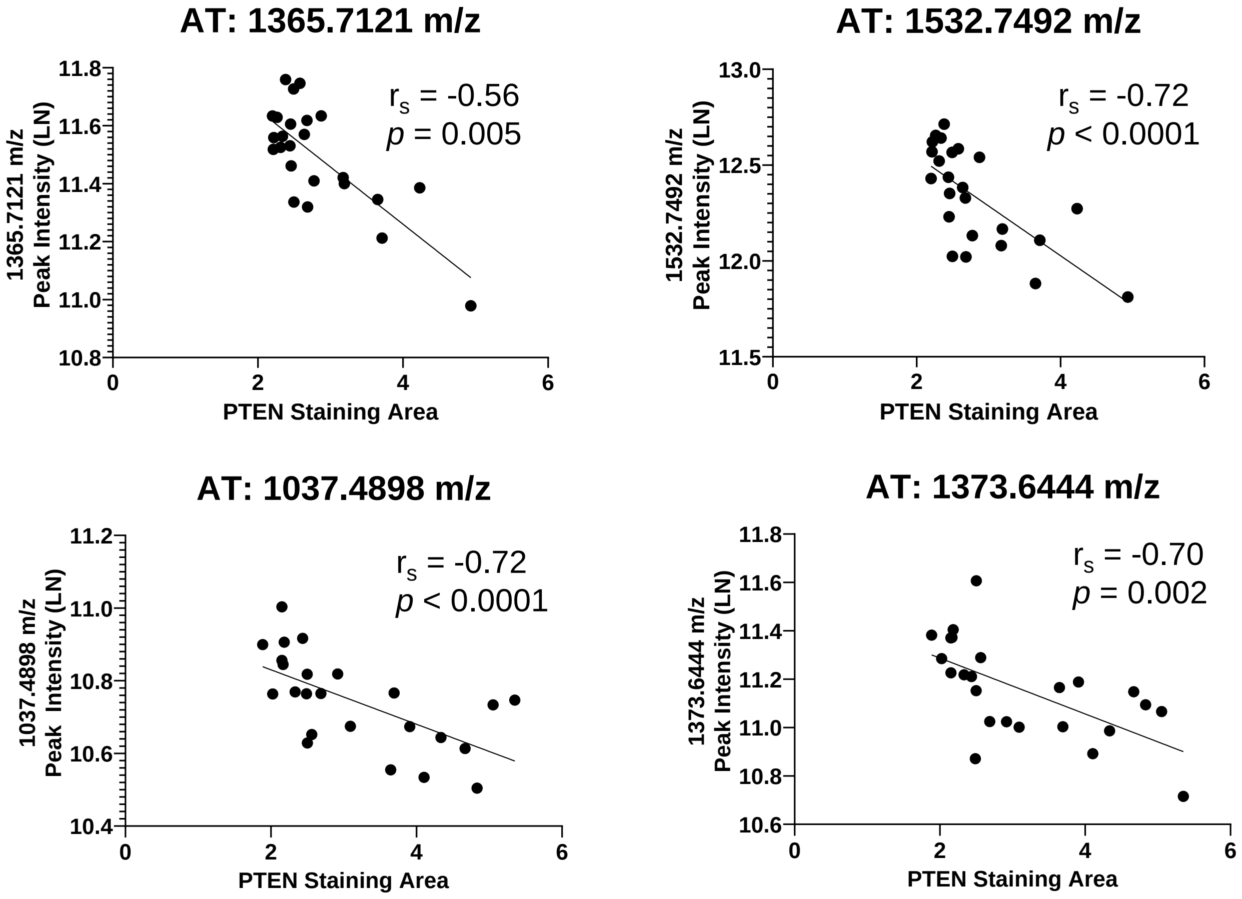

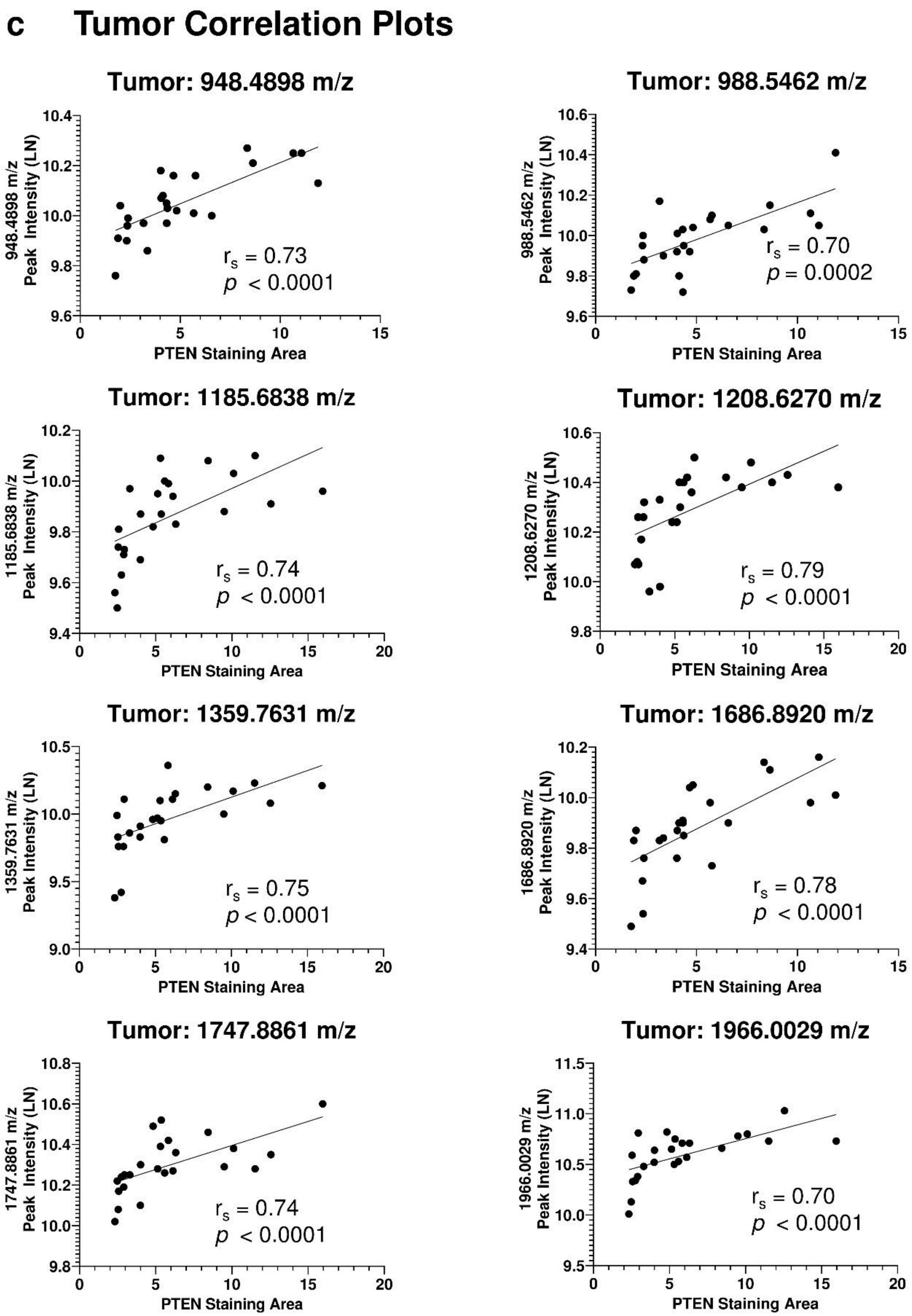

Figure S8. PTEN stained human breast TMA datasets demonstrate significant correlation between PTEN staining area and peak intensity. PTEN (1:200) staining area of breast (**a**) normal adjacent to the tumor (NAT), (**b**) adjacent to the tumor and (**c**) malignant tumor (Tumor) TMAs correlated to TMAs peak intensity (LN). Spearman's rank-order correlation coefficient (r_s) was calculated. All correlation plots generated a significant *p*-value (alpha =0.05).

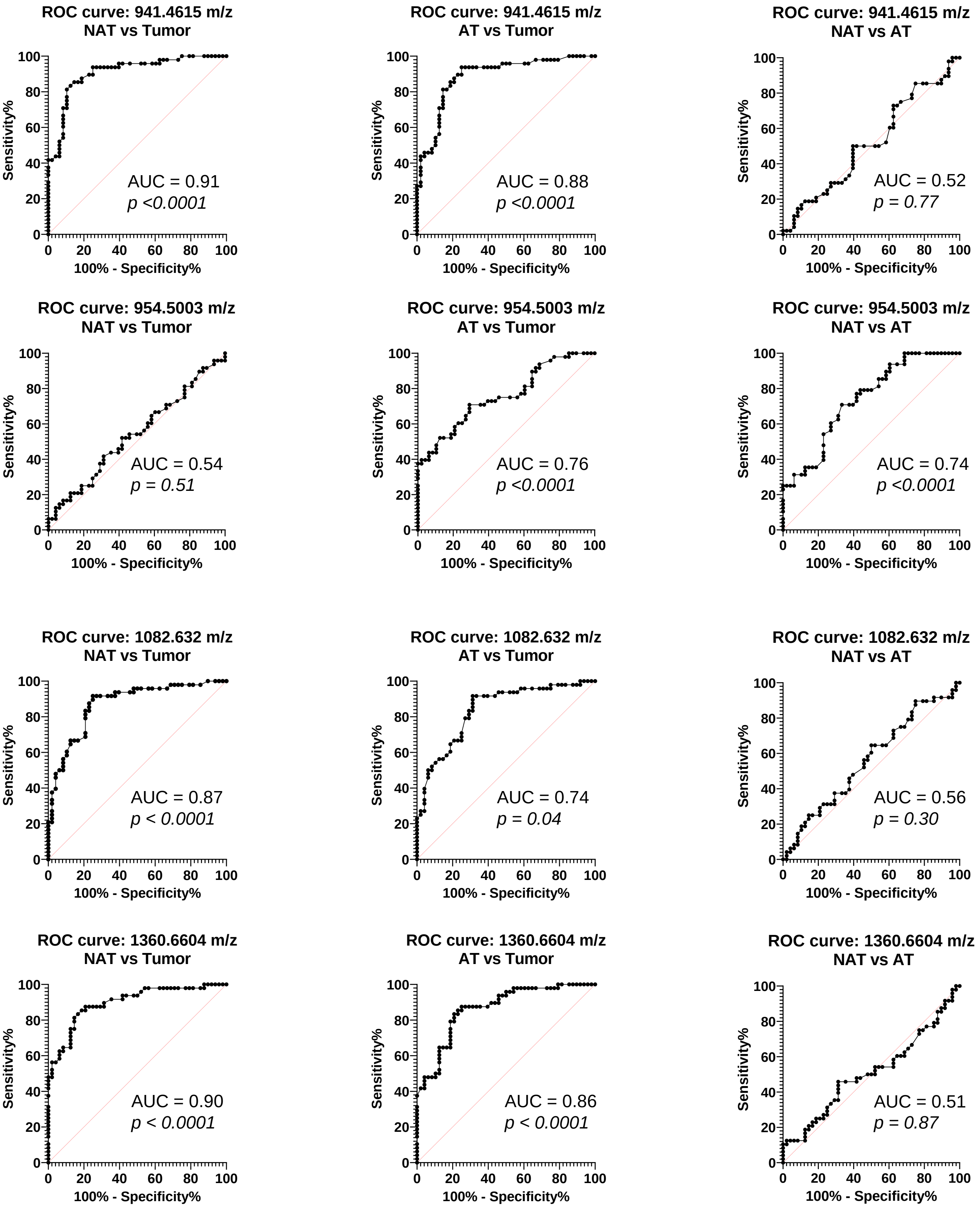

Figure S9. ROC analysis of PTEN stained human breast TMA datasets. Receiver Operating Characteristic (ROC) curve with AUC values are presented for selected peaks (AUC ≥ 0.70).

Table S1. List of peaks used for evaluation of reproducibility per consecutive tissue section.

Peaks (m/z)	882.4414	1041.5211	1302.6330	1777.8063
733.3183	883.3848	1045.5195	1321.5145	1829.8096
735.3217	884.4211	1054.4243	1324.6119	1861.7126
740.3936	887.3788	1058.5070	1328.5787	1942.7367
741.3298	890.4004	1062.4464	1343.6457	1964.7960
757.6577	893.3784	1068.4883	1350.5803	2018.8257
767.3544	897.4368	1074.5992	1359.6519	2050.7307
774.4187	900.4151	1082.6208	1365.6316	2096.8844
781.3150	905.3883	1097.4657	1386.6165	2155.7664
785.3880	909.3667	1098.5674	1397.5365	
791.3359	913.4033	1104.5956	1399.6164	
795.3216	914.4811	1108.4472	1406.6655	
797.4220	919.4357	1115.5315	1410.6382	
797.4238	921.3945	1125.5167	1421.5888	
801.3841	927.3714	1128.4992	1426.6206	
804.3686	928.4610	1131.5189	1432.5913	
809.4584	935.3652	1135.4894	1437.6099	
812.3956	938.4669	1137.5138	1438.6465	
815.3597	941.4443	1139.5599	1444.5856	
819.4002	944.4852	1140.4451	1458.6653	
823.3721	954.3947	1142.4873	1472.6318	
827.4054	954.4880	1147.5007	1480.6623	
828.4341	963.4208	1150.4948	1483.7040	
834.4005	970.5046	1159.5268	1491.6157	
836.3600	976.4852	1161.5669	1505.6268	
839.3322	984.4297	1164.4584	1533.6394	
840.3654	990.4085	1172.5076	1540.7388	
841.4168	996.4039	1179.5197	1570.6458	
843.3622	1001.5022	1188.5376	1588.7778	
844.3935	1003.4084	1194.4698	1592.6244	
847.4643	1008.4407	1201.5200	1609.7334	
849.4304	1012.3902	1211.5775	1611.5796	
856.3027	1016.4221	1230.4933	1621.6096	
857.3639	1017.4588	1233.5743	1629.6666	
858.4053	1019.5040	1242.5384	1651.6920	
863.3883	1021.4504	1243.4412	1669.7457	
865.3973	1032.4630	1257.5352	1672.6691	
869.4372	1034.4178	1264.5189	1675.7038	
870.3907	1034.5630	1280.6580	1681.7595	
872.4493	1039.4466	1283.5849	1767.8501	
875.3868	1040.4589	1286.6307	1772.6750	
